# Comparative proteomics of the vector *Dermacentor reticulatus* revealed differentially regulated proteins associated with pathogen transmission in response to laboratory infection with *Rickettsia slovaca*

**DOI:** 10.1186/s13071-019-3564-y

**Published:** 2019-06-24

**Authors:** Gabriela Flores-Ramirez, Balázs Sallay, Maksym Danchenko, Olha Lakhneko, Eva Špitalská, Ludovit Skultety

**Affiliations:** 1 Institute of Virology, Biomedical Research Center, Slovak Academy of Sciences, Dúbravská cesta 9, 845 05 Bratislava, Slovak Republic; 2 Institute of Microbiology of the Czech Academy of Sciences, v.v.i., Videnska 1083, 142 20 Prague, Czech Republic

**Keywords:** TIBOLA, Tick vector, Blood-feeding, Comparative proteomics, Protective antigens, Immune modulation, Bacterial transmission

## Abstract

**Background:**

Tick-borne rickettsial diseases are caused by pathogens acquired from hard ticks. In particular, *Rickettsia slovaca*, a zoonotic infectious bacterium causing tick-borne lymphadenopathy (TIBOLA), is transmitted by the vectors *Dermacentor* spp. that can be found all over Europe. Although recent studies point out the extreme complexity of bacteria-induced effects in these blood-feeding vectors, the knowledge of individual molecules involved in the preservation and transmission of the pathogen is still limited. System biology tools, including proteomics, may contribute greatly to the understanding of pathogen-tick-host interactions.

**Methods:**

Herein, we performed a comparative proteomics study of the tick vector *Dermacentor reticulatus* that was experimentally infected with the endosymbiotic bacterium *R. slovaca*. *Rickettsia*-free ticks, collected in the southern region of Slovakia, were infected with the bacterium by a capillary tube-feeding system, and the dynamics of infection was assessed by quantitative PCR method after 5, 10, 15 and 27 days.

**Results:**

At the stage of controlled proliferation (at 27 dpi), 33 (from 481 profiled) differentially abundant protein spots were detected on a two-dimensional gel. From the aforementioned protein spots, 21 were successfully identified by tandem mass spectrometry.

**Conclusions:**

Although a few discovered proteins were described as having structural or housekeeping functions, the vast majority of the affected proteins were suggested to be essential for tick attachment and feeding on the host, host immune system evasion and defensive response modulation to ensure successful pathogen transmission.

**Electronic supplementary material:**

The online version of this article (10.1186/s13071-019-3564-y) contains supplementary material, which is available to authorized users.

## Background

Ticks are obligate haematophagous ectoparasites of a wide range of vertebrate hosts, including humans and other mammals, which are invading new territories due to global climate change. They are considered one of the most important vectors of zoonotic diseases and epidemics caused by viruses, bacteria and protozoans [1, 2]. These pathogens represent a serious public health risk as well as an undesirable source of economic loss due to reduced meat and milk production or impaired reproductive function in livestock, including late-term abortions and stillbirths [2]. Thus, it is essential to increase protection against these vectors, which represent a significantly growing burden on human and animal health worldwide.

*Dermacentor reticulatus* (Fabricius, 1794) is a widely and abundantly spread tick species in Europe [3–5]. It has a high reproduction rate, and is very resistant against unfavourable environmental conditions [4, 5]. This species is responsible for transmitting various tick-borne pathogens: (i) viruses, e.g. Omsk haemorrhagic fever virus or tick-borne encephalitis virus; and (ii) bacteria, e.g. *Rickettsia slovaca* (tick-borne lymphadenopathy agent, TIBOLA), *Coxiella burnetii* (responsible for Q fever) and *Francisella tularensis* (the causative agent of tularemia). These ticks also represent a major vector for blood parasites such as *Babesia canis* (responsible for canine babesiosis), *Babesia cabalii* and *Theileria equi* (agent of equine piroplasmosis) [4–6].

*Rickettsia slovaca* is a spotted fever group rickettsia that was isolated in 1968 for the first time from the tick *Dermacentor marginatus* collected in central Slovakia [7]. Subsequently, it has been detected or isolated from *D. marginatus* and *D. reticulatus* throughout Europe [8–14]. The bacterium has a typical rod shape with a diameter of 0.37–0.45 μm and a length of 0.8–1.2 μm [15]. It is a causative agent of the mild human disease TIBOLA, which has been confirmed in many European countries. The infection is accompanied by tick bite-related skin lesions and cervical lymphadenopathies [13, 14, 16–20].

Next-generation sequencing showed considerable diversity of the tick inner microbiome [21]. Due to evolved control mechanisms, it is plausible to regulate the proliferation of the commensal microbes without causing a notable impact on the fitness of the vector [22]. Tick innate immunity is based on the coordinated action of humoral and cellular immune responses. The invading microbes are phagocytosed by tick haemocytes, which are navigated by the primordial complement-like system composed of thioester-containing proteins, fibrinogen-related lectins and convertase-like factors. The midgut is a major organ where the microbes that are ingested *via* the blood meal encounter the vector’s internal tissues. Direct antimicrobial action is carried out by a variety of specialised molecules including defensins, lysozymes, microplusin, hebraein, 5.3 kDa family polypeptides, reactive oxygen species, etc. However, this dynamic process is more complex. Apart from the vector and host immune response, the bacterial symbionts and pathogens also have their own molecular tools to manipulate the defence response in order to induce infection in both tick and mammalian host.

Recently, advancements in the understanding of tick-pathogen interaction have been achieved by several transcriptomics or proteomics studies. To verify the hypothesis that the tick immune system may control the preservation of pathogens, Jaworski et al. [23] traced the expression of *Dermacentor variabilis* tick genes induced by a bacterial infection. Adjustments in the expression of genes, which are likely encoding tick immune-related proteins, clearly demonstrated the complexity of the defence system of arachnids. Further proteomics studies allowed identifying novel secreted proteins in *Dermacentor andersoni* [24] as well as revealing molecules associated with the adaptive stress response to rickettsial infection in unfed *D. reticulatus* larvae [6]. Furthermore, Rachinsky et al. [25] established a proteome database containing proteins involved in successful pathogen transmission. Among others, proteins implicated in signalling processes were discovered using comparative proteomics [25, 26]. Interestingly, it has also been reported that proteins from apoptotic signalling pathways of *Ixodes scapularis* were more heavily regulated in the midguts than in salivary glands in response to *Anaplasma phagocytophilum* infection [27].

Indeed, the application of proteomics seems to be essential for understanding the intimate details in tick-host-pathogen interaction. New discoveries related to vector competence and biological processes associated with the transmission of tick-borne diseases are needed. Furthermore, proteomics studies might even result in the identification of candidate protective antigens that are capable of blocking the transmission of pathogens. Identifying these proteins may lead to the development of protective vaccines [28, 29] aiming to reduce the vector capacity for tick-borne pathogens. In this study, we investigated the differences in the abundance of *D. reticulatus* proteins in response to *R. slovaca* infection.

## Methods

### Collecting the questing ticks

Questing *D. reticulatus* adult ticks were collected in Gabčíkovo during 2016 and 2017. The locality (47°53′N, 17°32′E) is situated in southwestern Slovakia, at 110 m above sea level. Ticks were collected in an alluvial habitat near the River Danube by dragging a woollen flag over the lower vegetation.

Collected ticks were rinsed with 70% ethanol and sterile water and then dried. Subsequently, a small part of the leg was excised from each individual. Total genomic DNA was isolated by boiling the legs in 0.7 M ammonium hydroxide [30] and analysed by PCR. Female ticks that tested negative for *Rickettsia* spp. were split into two (infected and control) groups and kept in the laboratory during the experiment.

### Laboratory infection of ticks

*Rickettsia slovaca* strain 13-B, originating from the collection of the Department of Rickettsiology (Institute of Virology, Biomedical Research Center, Bratislava, Slovakia) was revived and propagated in Vero cell line (*Cercopithecus aethiops* monkey epithelial cells ATCC CCL-81) in Dulbecco’s modified Eagle’s medium (DMEM), supplemented with 3% fetal bovine serum (FBS) at 34 °C.

**Fig. 1:**
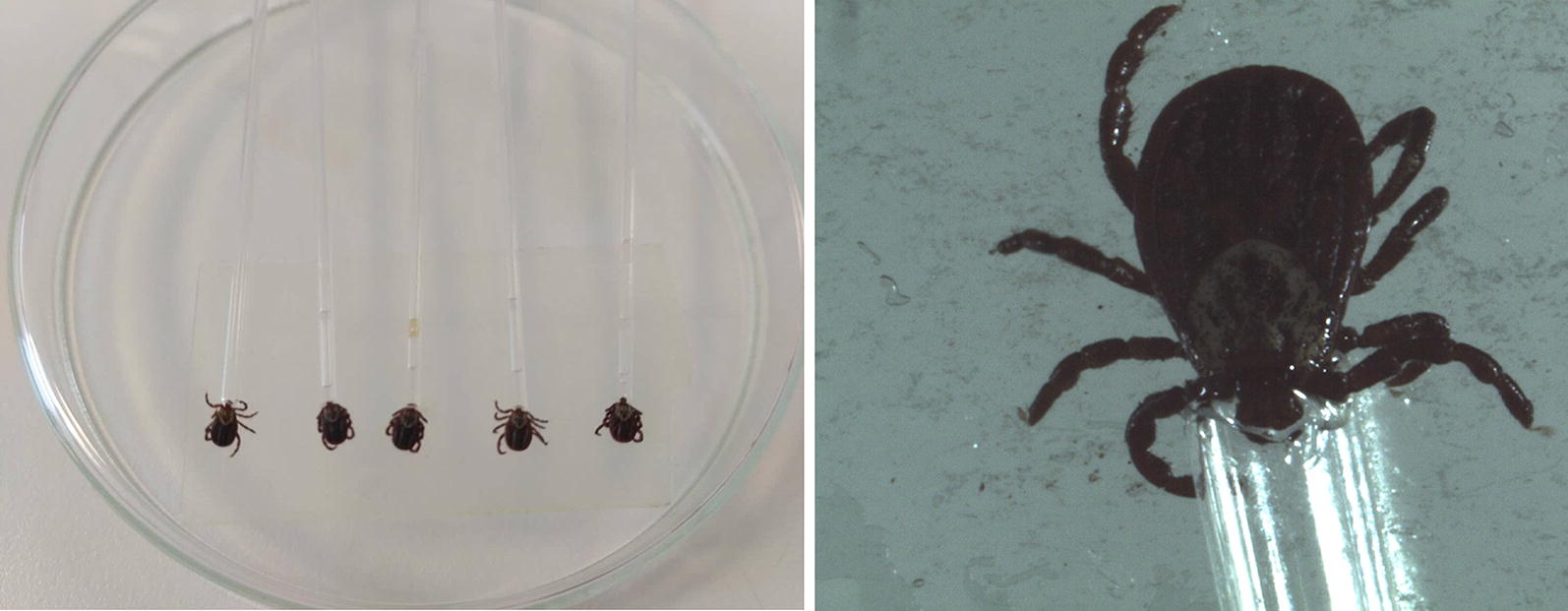
Laboratory infection of *D. reticulatus* with *R. slovaca* by capillary tube feeding method

*Rickettsia* spp.-negative ticks divided into three biological replicate groups of five ticks (per time point) were infected with the bacterium by capillary tube feeding (infected groups) (Fig. 1). Briefly, the drawn-out glass microcapillary pipettes were loaded with approximately 2 μl of the medium containing 10^4^/μl *R. slovaca* (assessed by quantitative real-time PCR described below). The pipettes were then placed over hypostomes of ticks immobilised in Petri dishes as described previously for infection with *Borrelia* and *Rickettsia helvetica* [31, 32]. The ticks were then allowed to feed for 1 h at room temperature (RT). As a control group, *Rickettsia* spp.-negative ticks were fed bacteria-free medium (control group).

### Protein and complete RNA extractions and cDNA synthesis

After 5, 10, 15 and the 27 days post-infection (dpi), the ticks from both the infected and the control group were frozen in liquid nitrogen and ground down to a fine powder using a mortar and a pestle. Subsequently, genomic DNA, complete RNA and total protein were extracted using a DNA/RNA/Protein Mini Kit (Qiagen, Hilden, Germany), following the instructions of the manufacturer. Samples were stored at − 80 °C until further use.

The fraction containing RNA was treated with RQ1 RNA-free DNAase (Promega, Madison, USA) for 4 h in order to eliminate possible contamination with genomic DNA. The samples were then cleaned by a RNAeasy miniElute cleanup kit (Qiagen) and spectrophotometrically quantified by a NanoDrop 2000 (Thermo Fisher Scientific, Wilmington, USA). Aliquots of 1 μg RNA isolated from the different conditions were amplified using a First Strand cDNA synthesis kit (Thermo Fisher Scientific, Carlsbad, USA), following the manufacturer’s instructions (product stored at − 20 °C until used).

### Molecular identification of pathogens in ticks

Tick samples were screened prior the experiment by PCR-based methods for the presence of *Rickettsia* spp. Amplification of the *gltA* gene [33] was used for detection. Real-time PCR assays based on the *ompB* gene were applied for specific identification of *R. slovaca* [34] and other species (*Babesia* spp. (conventional PCR), *Coxiella burnetti* and *R. raoultii* [34–36]) presented in the samples to exclude concurrent infection. All primers and probes (Table 1) were synthesised by Microsynth AG (Balbach, Switzerland).

**Table 1:** List of primers and probes used for detection of bacteria in ticks

Target organism/gene	Primers and probes	Sequence (5′-3′)	Reference
*Rickettsia* sp./*gltA*	CS-F	TCGCAAATGTTCACGGTACTTT	[33]
	CS-R	TCGTGCATTTCTTTCCATTGTG	
	CS-P	FAM-TGCAATAGCAAGAACCGTAGGCTGGATG-BHQ	
*R. slovaca*/*ompB*	Rslo351F	CAGGTCAAGGTATTACTAATGCAC	[34]
	Rslo479R	CACCGAAGTCTATGCTTCCTACAC	
	RsloP	FAM-TGTAATAATGGTGCTGCTATTGG –TAMRA	
*R. raoultii/ ompB*	Rraou2850F2	GTGGTGGTGTTCCTAATACTCC	[34]
	Rraou2956R2	ACCTAAGTTGTTATAGTCTGTAGTAAAC	
	Rrao2896P	6-FAM-CGCGATATTGGCACTGTACAGT TAAAGCATCGCG-TAMRA	
*Babesia* sp./*18S* rRNA	BJ1	GTCTTGTAATTGGAATGATGG	[35]
	BN2	TAGTTTATGGTTAGGACTACG	
*Coxiella burnetii*/*16S* rRNA	CbP1	GGGAAACTCGGGCTAATACC	[36]
	CbP2	CACGAGGTCCGAAGATCC	
	CbP	FAM-CCCGCTTTGCTCCAAAGAGATTATG-TAMRA	

The real-time PCR mixtures contained 10 μl of the 2× SuperHot Master mix (Bioron, Ludwigshafen, Germany), 100 nM of each primer, 100 nM probe, 0.5 μl of 100 mM MgCl_2_ and 3 μl of the template DNA/cDNA, the total volume being 20 μl. The PCR programme consisted of an initial denaturation at 95 °C for 3 min, followed by 40 cycles of denaturation at 95 °C for 20 s and annealing at 60 °C for 40 s. The real-time PCR assays were carried out using a CFX96™ Real-Time System (Bio-Rad, Hercules, USA). The DNA from rickettsiae-negative ticks and nuclease-free water were used as negative controls in each reaction. Genetic material from *R. slovaca*, *R. raoultii* [9] and *Babesia canis* purified from ticks as well as the in-house reference strains (*R. slovaca*, *C. burnetii*) were used as positive controls. The analyses were performed in two technical replicates.

**Fig. 2:**
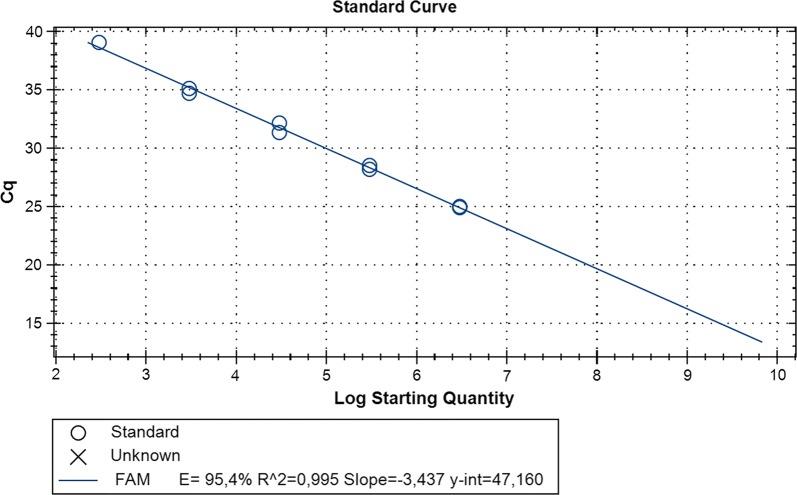
Standard calibration curve for quantification of the rickettsial *ompB* gene showing the linear relationship between threshold cycle (Cq) values and log starting quantity for serially diluted standard

For absolute quantification of *R. slovaca* in capillary-fed ticks, the standard curve was generated as described by Jiang et al. [34]; the limit of detection for qPCR assay was determined at 3 × 10^2^ copies of the target gene (Fig. 2). A one-way ANOVA calculator was used to compare copy numbers in each time point throughout the study (http://www.socscistatistics.com). The significance threshold was set at *P* = 0.05.

### Protein separation and quantification by gel electrophoresis

Total tick protein was obtained after the RNA extraction step of the DNA/RNA/Protein Mini Kit (see above). The precipitated proteins were reconstituted in a buffer which contained 7 M urea, 2 M thiourea, 40 mM Tris, 1% amidosulfobetaine-14 and 1% Triton X-100; the protein concentration was determined by a 2-D Quant Kit (GE Healthcare, Diegem, Luxembourg). For initial screening, polyacrylamide gel electrophoresis was performed in the presence of sodium dodecyl sulphate (SDS-PAGE). Briefly, aliquots containing 15 μg of solubilised proteins were boiled for 5 min, and the volume was adjusted with 4× Laemmli buffer (8% SDS, 240 mM Tris, pH 6.8, 40% glycerol, 2% 2-mercaptoethanol and 0.04% bromophenol blue). Separation was carried out in 15% polyacrylamide gels with Tris-glycine running buffer, pH 8.3, using Protean Tetra mini Cell apparatus (Bio-Rad) at 60 V for 15 min, followed by 200 V until the tracking dye reached the bottoms of the gels. After separation, the proteins were visualised by staining with colloidal Coomassie Blue G-250 [37]. Several apparently different bands were excised manually and digested by trypsin as described below.

At 27 dpi, control group and infected group protein samples, prepared in triplicates, were used for quantitative comparison using 2-DE. Protein aliquots of 150 μg were adjusted to 340 μl with isoelectric focusing buffer (7 M urea, 2 M thiourea, 40 mM Tris 1% amidosulfobetaine-14, 1% Triton X-100, 1% ampholytes pH 3–10 and 1% DeStreak). After centrifugation at 10,000× *g* for 10 min, the supernatant was transferred to 18 cm immobilised non-linear pH gradient strips 3–10 (GE Healthcare). Following overnight passive rehydration, first-dimensional separation was carried out using an Ettan IPGphor 3 unit (GE Healthcare). The strips were reduced for 15 min in 4 ml of equilibration buffer (0.1 M Tris pH 6.8, 30% glycerol, 6 M urea and 3% SDS) containing 2% dithiothreitol and then alkylated in the dark for 15 min in the same buffer with 2.5% iodoacetamide. After subsequent washing in running buffer (25 mM Tris, 192 mM glycine and 0.1% SDS), the strips were placed on top of 13% polyacrylamide gels and sealed with 0.5% agarose containing 0.002% bromophenol blue in the running buffer. The second-dimensional separation was carried out in Protean II xi Cell (Bio-Rad) at 10 mA per gel for 1 h and 30 mA per gel until the tracking dye disappeared. The gels were fixed two times for 30 min in 50% methanol containing 7% acetic acid and then stained in the dark overnight with SYPRO Ruby (Invitrogen, Carlsbad, USA). This was followed by two washing steps for 30 min, the first in 10% methanol with 7% acetic acid and the second in milliQ H_2_O. The gels were then scanned on a PharosFX Molecular Imager (Bio-Rad) and stained in colloidal Coomassie blue for manual excision of target spots [37].

Quantitative software-assisted gel analysis was performed using SameSpots v.5.1 software (TotalLab, Manchester, UK). The images were aligned, and dust particles, stain particles, streaks or damaged gel areas were filtered out. The protein spots were then manually validated, and the determined relative volumes were normalised to the median distribution of reference gel in order to compensate minor differences in sample loading. Differentially abundant protein spots found in the infected and control samples were chosen based on an ANOVA *P*-value ≤ 0.05 and an abundance ratio ≥ 1.5.

### In-gel protein digestion

The excised protein spots were washed with agitation in 50 mM ammonium bicarbonate (ABC; Sigma-Aldrich, Darmstadt, Germany) containing 50% acetonitrile (ACN; Merck, Darmstadt, Germany) at RT. After complete destaining, the gel pieces were dehydrated for 10 min at RT in 100% ACN, reduced for 30 min at 50 °C with 10 mM dithiothreitol (DTT), and alkylated for 30 min in the dark with 50 mM iodoacetamide in 100 mM ABC. The gel plugs were then rewashed three times with 50 mM ABC for 10 min at RT, dehydrated with 100% ACN, and incubated for 14 h at 37 °C in digestion solution (40 ng of lyophilised sequencing grade modified trypsin (Promega) per 5 µl of 10 mM ABC and 10% ACN). The resulting peptide mixtures were extracted twice by 50 µl of 1% trifluoroacetic acid in 70% ACN. The total volume of samples was reduced to 20 µl by vacuum evaporation using a Concentrator plus (Eppendorf, Hamburg, Germany). The solution was then stored at − 20 °C.

### Mass spectrometry and bioinformatics for protein identification

The peptides were analysed by LC-MS/MS using nanoAcquity UHPLC (Waters, Manchester, UK) and Q-TOF Premier (Waters). Briefly, the samples were loaded onto the trap column and desalted. Subsequently, they were separated on a BEH130 C18 analytical column (200 mm length, 75 μm diameter, 1.7 μm particle size), using a 20 min gradient of 5–40% ACN containing 0.1% formic acid at a 300 nl/min flow rate. The column outlet was connected to the PicoTip emitter (360 μm outer diameter, 20 μm inner diameter, 10 μm tip diameter) and the eluted peptides were nanosprayed (3.4 kV capillary voltage) to the mass spectrometer. Spectra were recorded in the MSE mode (parallel high and low energy traces without precursor ion selection). Ions with 50–1950 m/z were detected in both channels. The external mass calibrant Glu1-Fibrinopeptide B (500 fmol/ml) was infused through the reference line at a 500 nl/min flow rate and used for mass correction.

Data were processed by ProteinLynx Global Server v.3.0 (Waters). For peak picking, the following threshold parameters were applied: low energy 140 counts and high energy 30 counts. Precursors and fragment ions were coupled, using correlations of chromatographic elution profiles in low/high energy traces. Spectra were searched against *Ixodidae* protein sequences supplemented with *R. slovaca* and *R. raoultii* proteomes downloaded from UniProt in June 2017 (137,542 entries, http://www.uniprot.org) and BLAST-ed against the SwissProt database (http://www.uniprot.org/, containing 545,536 entries downloaded in June 2017). Workflow parameters for the protein identification searches were: (i) maximum one possible trypsin miscleavage; (ii) a fixed modification (carbamidomethyl cysteine) and a variable modification (oxidised methionine and deamidated asparagine or glutamine); (iii) the mass tolerance of precursors and fragments was automatically determined by the software; and (iv) peptide matching was limited to less than 4% false discovery rate against the randomised database. Identifications were accepted whenever at least two distinct reliable peptides matched the protein sequence. The protein localisations were predicted with PSORT algorithm (https://psort.org).

## Results

### Confirmation of successful infection of ticks

**Fig. 3:**
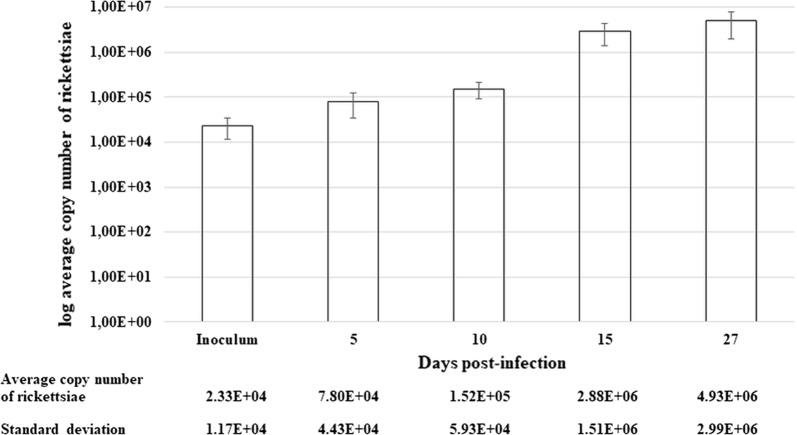
Growth kinetics of *R. slovaca* in *D. reticulatus* after laboratory infection. Shown are the mean numbers of log copy of the rickettsial *ompB* gene in ticks on the day 5, 10, 15 and 27 post-infection. The bars represent the standard deviation of the mean for three biological replicates

The collection of ticks was performed by dragging a woollen flag over the lower vegetation in Gabčíkovo (southern Slovakia). Subsequently, *D. reticulatus* ticks were analysed for rickettsial infection using PCR. The *Rickettsia*-negative ticks were infected with *R. slovaca* by the capillary tube feeding method, and the presence of the bacterium was assessed after 5, 10, 15 and 27 days post-infection (dpi) by quantitative PCR (qPCR). This analysis confirmed the successful infection of ticks with the bacterium, showing a range of 7.6 × 10^4^ to 4.9 × 10^6^ average copy numbers (*P* < 0.05) (Fig. 3). All samples were PCR-negative for *R. raoultii*, *Babesia* spp. and *Coxiella burnetii.*

### Monitoring the response to infection by SDS-PAGE

**Fig. 4:**
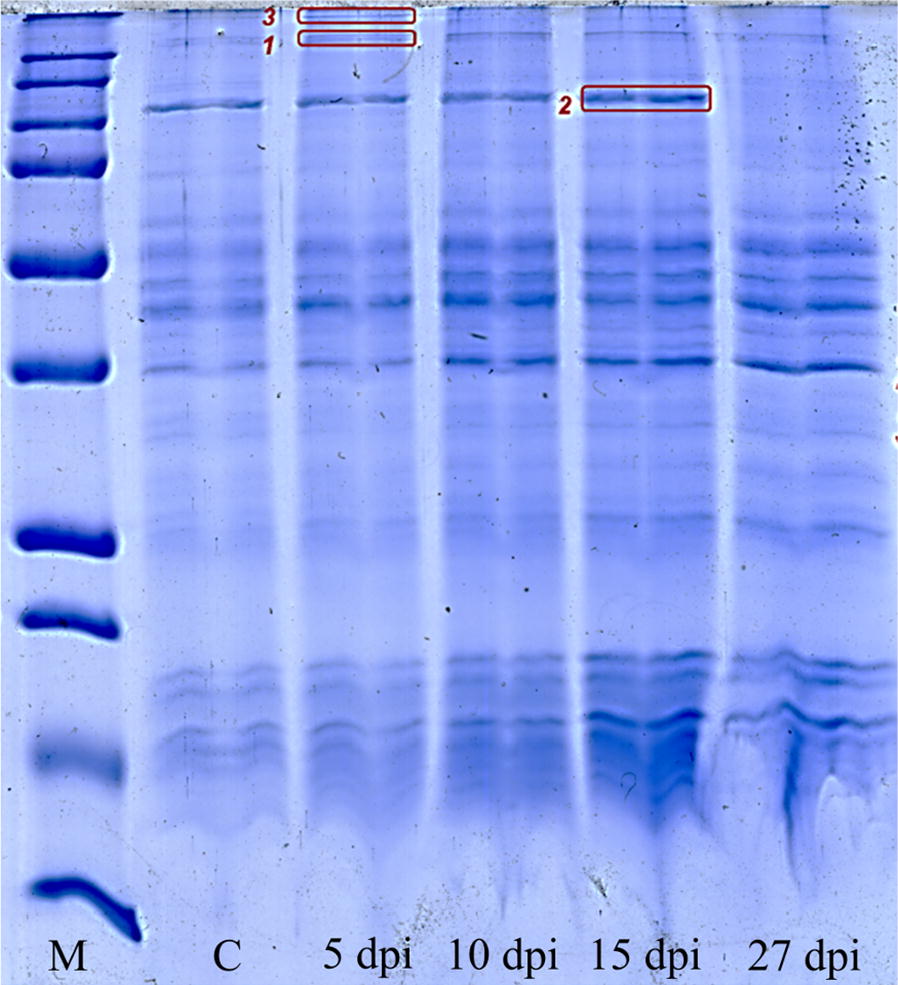
SDS-PAGE protein pattern over the course of time of *R. slovaca* infected *D. reticulatus*. *Abbreviations*: M, protein molecular weight markers; C, control

The ticks were frozen in liquid nitrogen, ground in a mortar, and subjected to lysis followed by protein extraction and separation of the mixtures by SDS-PAGE (Fig. 4) after 5, 10, 15 and 27 dpi. In comparison with the control group, the three bands that appeared differentially abundant were selected and excised. Proteins present in the gel pieces were digested by trypsin and identified by tandem mass spectrometry. The abundance of band #1, containing an unknown tick host protein, increased gradually due to the presence of *R. slovaca*. The main protein in band #3 was identified as paramyosin, an immunomodulatory protein playing an important role in immune reactions against parasites in vertebrate hosts [38]. Finally, the major protein in band #2 was recognised as putative defensin. This protein was present in practically all samples, including the control ones (in lower abundance). However, it almost completely disappeared from the sample collected at 27 dpi.

### Tick-pathogen interaction during “steady-state” infection at 27 dpi

**Fig. 5:**
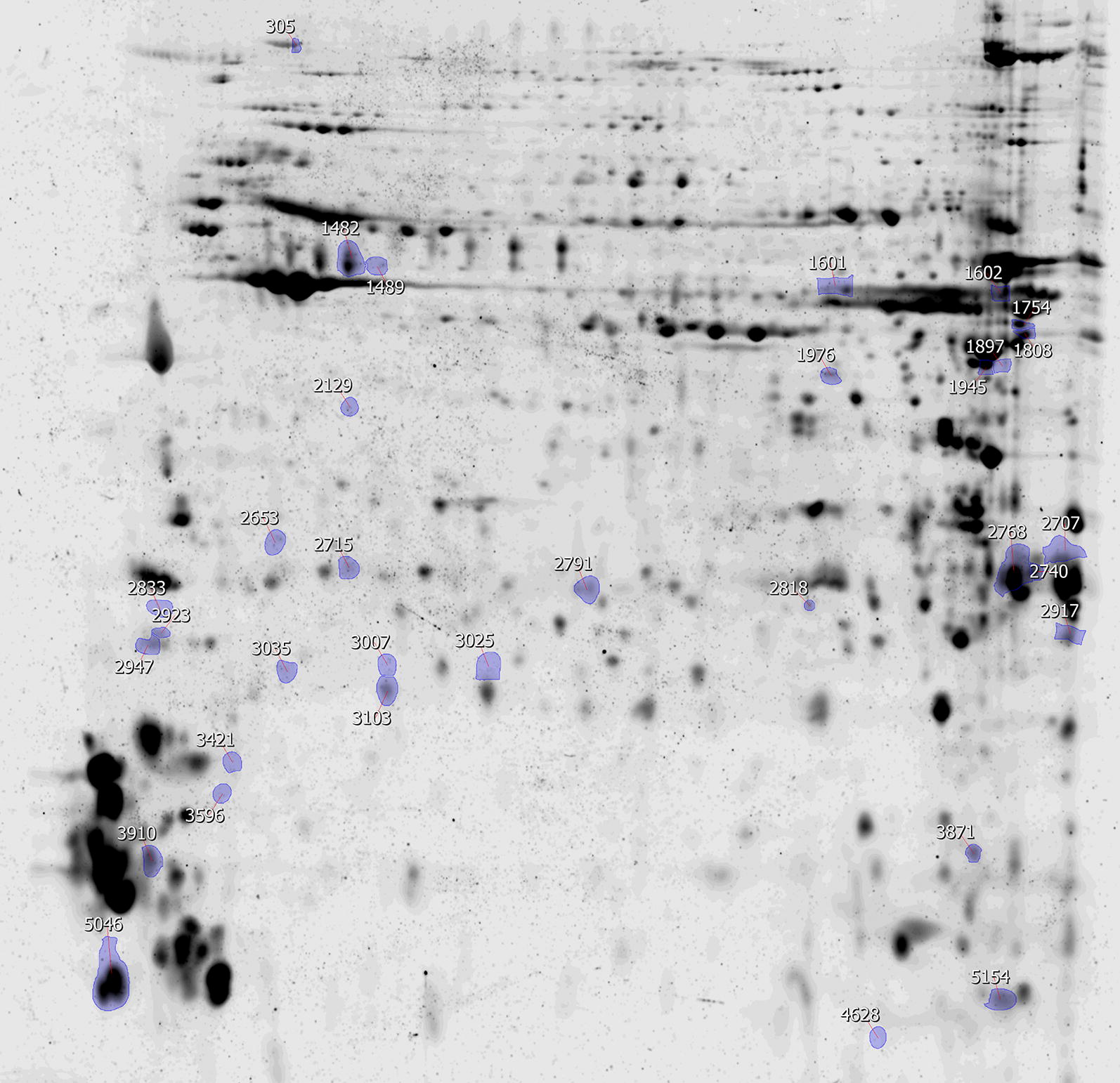
Representative gel image from the control set with 33 annotated differentially abundant protein spots

To reveal the intimate details of the tick-host-pathogen interaction and to figure out how the tick immune system maintains the presence of pathogens during “steady-state” infection, we tracked changes in the protein abundance of *D. reticulatus* tick proteins compared to control, during the “steady-state” rickettsial infection at the latest infection time point (27 dpi). After two-dimensional gel electrophoresis (2-DE) (Fig. 5 and Additional file 1: Figure S1) was performed; 481 reproducibly quantified gel spots were detected. All data, including descriptive statistics, are shown in Additional file 2: Table S1. Comparison with the control group of samples discovered 33 (6.9%) differentially abundant spots (Fig. 5), 21 of which were successfully identified by LC-MS/MS (Table 2). Following the acquisition of relevant functional information, we described 6 secreted proteins, 5 proteins functioning in the cytoplasm, 4 nuclear proteins, 2 proteins localised in mitochondria and 1 unknown function protein, which is predicted to have a membrane localisation (Table 2). Two gel spots (spots 1482 and 1489) that were more abundant in response to *R. slovaca* challenge at 27 dpi were identified as salivary serine protease inhibitors, serpins; one spot (spot 1976) as a zinc-binding oxidoreductase and one spot as a putative secreted protein (ISCW gene, spot 3035). We discovered three gel spots that corresponded with secreted glycine-rich (GRP; spots 1897 and 1945) and glycine-proline rich (spot 5154) proteins that were accumulated in response to the infection. Troponin I-like protein (spot 2707), glutathione S-transferase (GST; spot 2818), heat-hock protein 20 (HSP20; spot 2715) and a putative ML-domain protein (spot 2791) were more abundant in response to rickettsial infection.

**Table 2:** List of identified differentially abundant tick proteins, showing spot number, UniProt identifier, protein name, function annotated from BLAST similarity search, localisation assigned by PSORT algorithm, ProteinLynx reliability score, number of matched peptides and sequence coverage, theoretical and experimental molecular weights/isoelectric points, and if the protein is more abundant (↑) or less abundant (↓) upon *Rickettsia* infection

Spot	Accession	Description	Function	Localisation	PLGS score	Peptides/ coverage (%)	Theoretical MW (kDa)/pI	Experimental MW (kDa)/pI	Regulation
1482	L7LTU1	Put. tick salivary serpin, *Rhipicephalus pulchellus*	Serin protease inhibitor	Secreted	417	9/17.5	44/5.8	48/5.5	↑
1489	L7LTU1	Put. tick salivary serpin, *Rhipicephalus pulchellus*	Serin protease inhibitor	Secreted	711	6/12.0	44/5.8	48/5.7	↑
1897	L7LRE8	Put. glycine rich protein, *Rhipicephalus pulchellus*	Salivary gland peptide	Secreted	7161	15/24.9	38/9.0	42/9.5	↑
1945	L7LRE8	Put. glycine rich protein, *Rhipicephalus pulchellus*	Salivary gland peptide	Secreted	3053	14/24.9	38/9.0	41/9.3	↑
2791	A0A131XM62	Put. ML domain protein, *Hyalomma excavatum*	Salivary lipid interaction	Secreted	969	3/14.8	20/7.9	24/7.0	↑
5046	A0A131XS44	Secreted protein, *Hyalomma excavatum*	Cuticle	Secreted	531	4/19.5	14/4.7	14/4.1	↓
5154	A0A023FST8	Put. large gyy 1, glycine-proline rich *Amblyomma cajennense*	Salivary gland peptide	Secreted	4046	5/29.8	14/9.8	14/9.5	↓
3035	B7QNW6	Put. secreted protein, *Ixodes scapularis*	Immunomodulation	Secreted	138	2/21.4	24/8.1	21/5.1	↑
1601	G3MND3	Phosphoglycerate kinase, *Amblyomma maculatum*	Glycolysis	Cytoplasm	2573	15/33.7	44/8.2	47/8.5	↑
1602	A0A131XNT2	Put. ubiquinone oxidoreductase ndufa9/39 kda sb., *Hyalomma excavatum*	Mitochondrial complex	Mitochondrion	5391	13/27.9	44/9.2	47/9.5	↑
1808	A0A147BGN0	Put. eukaryotic translation initiation factor 4 γ, *Ixodes ricinus*	Translation initiation factor	Cytoplasm/ nucleus	112	3/41.8	17/4.7	44/9.6	↓
1976	L7M642	Put. zinc-binding oxidoreductase, *Rhipicephalus pulchellus*	Quinone oxidereductase/ETC	Cytoplasm	421	3/12.7	36/8.7	41/8.4	↑
2715	A0A023FRH0	Put. heat shock protein, *Amblyomma cajennense*	HSP20 domain containining	Cytoplasm/ nucleus	403	3/11.9	22/6.2	26/5.5	↑
2818	B7SP22	Put. glutathione S-transferase, *Dermacentor variabilis*	Detoxification	Cytoplasm	2052	9/36.3	26/8.2	24/8.3	↓
2707	A0A131YF71	Troponin I protein, *Rhipicephalus appendiculatus*	Muscle contraction	Cytoplasm	217	3/11.5	23/10.3	26/9.8	↑
305	A0A1E1X279	Put. myosin class I heavy chain, *Amblyomma aureolatum*	Actin filament binding	Cytoplasm/ nucleus	1397	28/16.4	222/5.7	100/5.2	↓
2833	A0A023FIT8	Put. myosin regulatory light chain, *Amblyomma cajennense*	Muscle contraction	Cytoplasm	159	4/17.1	20/4.2	24/4.4	↑
2917	A0A131XKV8	Put. ATP synthase sb. o mitochondrial-like protein, *Hyalomma excavatum*	ATP synthesis/ proton transport	Mitochondrion	3087	10/23.7	23/10.1	23/9.9	↑
3025	B7Q9B4	Put. membrane protein, *Ixodes scapularis*	Porin	Cell membrane	227	2/51.2	9/9.7	21/6.3	↑
3871	A0A131YSI2	Ubiquitin-conjugating enzyme E2 L3, *Rhipicephalus appendiculatus*	Proteolysis	Cytoplasm/ mitochondrion	2337	7/55.8	18/9.1	17/9.3	↓
4628	A0A023GDJ7	Histone H4, *Amblyomma triste*	DNA binding	Nucleus	2630	11/60.2	11/11.8	13/8.7	↑

*Abbreviations*: put., putative; sb., subunit; ETC, electron transport chain

The putative myosin regulatory light chain (spot 2833) and the putative myosin class I heavy chain (spot 305) were regulated differently in infected vector *D. reticulatus*. Finally, we identified several differentially abundant proteins with housekeeping functions. The mitochondrial subunit of ATP synthase (spot 2917), ubiquinone oxidoreductase (spot 1602), phosphoglycerate kinase (spot 1601) and histone H4 (spot 4628) were accumulated in response to *R. slovaca* infection. On the other hand, an ubiquitin-conjugating enzyme E2 (spot 3871) and a putative eukaryotic translation initiation factor 4 γ (spot 1808) were less abundant in infected ticks. Apart from these housekeeping proteins, we also identified a structural constituent of the cuticle (spot 5046) and an uncharacterised protein that is an integral component of the membrane (spot 3025), which were either less or more abundant in infected ticks, respectively.

## Discussion

In Slovakia, tick vectors *D. reticulatus* and *D. marginatus* are generally infected with *R. slovaca* and *R. raoultii* [8, 9, 15, 39]. Gabčíkovo is a locality with a prevalence of *Rickettsia*-positive questing *D. reticulatus* adult ticks of 49.12% (95% CI: 36.14–62.10%). While *R. raoultii* was detected as a dominant species (45.61%, 95% CI: 32.68–58.54%), *R. slovaca*, *Coxiella burnetii* and *Babesia* spp. were found in only a few ticks (3.51%, 95% CI: 0–8.29%; 1.75%, 95% CI: 0–5.16%; 1.83%, 95% CI: 0.76–2.91%, respectively) [9]. In this study, *Rickettsia*-negative *D. reticulatus* ticks were successfully infected with *R. slovaca* by a capillary tube-feeding system, and the infection was maintained in the range of 7.6 × 10^4^ to 4.9 × 10^6^ copies throughout the duration of the experiment. These findings correspond with our previous result [39] in which we proved that the number of *R. slovaca* in *D. marginatus* increased from 1 × 10^4^ to 1 × 10^6^–4 × 10^6^ copies between 6 and 21 dpi. This observation may indicate tight control of *R. slovaca* proliferation with the tick vector.

Although, we realise that the ticks collected in the field might be subjected to an unknown random set of conditions including time elapsed since moulting, nutrient availability and the presence of other pathogens and symbionts, the identification of putative defensin (Fig. 4, band #2) in the preliminary SDS-PAGE analysis may indicate a response to an ongoing infection. Many of these molecules and their isoforms have been identified so far in both hard and soft ticks where they act either against Gram-positive or some Gram-negative-bacteria [40–48]. For instance, defensin-like peptide (DLP) from *Haemaphysalis longicornis* has been shown to have antimicrobial activity against drug-resistant microorganisms, including the fungus *Candida albicans* [49]. Interestingly, the putative defensin was detected in almost all samples in this study, including the control ones, although it was present in lower abundance. However, it almost completely vanished from the sample collected at 27 dpi. If we consider the antimicrobial activity of this molecule [49, 50], the observed findings may suggest a presence of “steady-state” infection with controlled bacterial proliferation.

Among the 21 identified proteins that were found to be differentially presented in response to *R. slovaca* challenge at 27 dpi, we recognised salivary serine protease inhibitors: serpins and their closely related [51] zinc-binding oxidoreductase. A similar result was reported by Ayllón et al. [27] and Liu et al. [52] who showed that the expression of protease inhibitors in salivary glands and midguts of adult female ticks was altered due to infection. Cystatins and serpins were also upregulated in *I. scapularis* that were infected with *A. phagocytophilum* and in *Ixodes ricinus* challenged with *Bartonella hensela.* On the other hand, several serpins, identified as components of the *D. variabilis* immune system, were found to be unregulated on a transcription level in response to bacterial infection [23]. This discrepancy, as shown by the results of these studies, can be explained by the fact that ticks produce numerous serpins. For instance, 45 serpins were detected in the genome of *I. scapularis*, 120 in *Amblyomma americanum* and 22 in *Rhipicephalus microplus*. There is also evidence of serpin expression, as 36 transcripts were detected in the transcriptome of *I. ricinus* and 10 in *Hyalomma excavatum* [53]. Despite numerous occurrences of these inhibitors in ticks, little is known about their biological function. It has been suggested that their key function is related to the modulation of the host immune system. Serpins, as well as a putative secreted protein (ISCW gene) [54] which was also accumulated in response to rickettsial infection, probably play a role in the mechanism that suppresses the defence reaction of vertebrate hosts. Apparently, it seems that *R. slovaca* possesses molecular tools for manipulating the defence response of the tick in order to induce an infection.

In this study, we also discovered glycine-proline rich proteins that accumulated in response to the infection. This corresponds with the result of Macaluso et al. [55], who showed that the genes encoding GRPs in *H. longicornis* were regulated upon tick infestation as a consequence of feeding. Indeed, these proteins were found to be essential for tick attachment and feeding on the host. Specifically, they were involved in the host’s immune system evasion [56]. Because the high expression of GRPs has been demonstrated in adult female ticks, these proteins have been proposed as potential candidate molecules in the formulation of an anti-tick vaccine cocktail for the biological control of ticks [56]. Likewise, a specific protective anti-tick immune response was shown in mice immunised by a recombinant troponin I-like protein from *H. longicornis* [57]. However, the main function of this protein is to regulate the contraction of striated muscle [58], and it has also been demonstrated to be expressed in salivary glands [59], where it can act as a potent inhibitor of angiogenesis [60]. Troponin I-like protein was found to be more abundant in our study in response to rickettsial infection.

Detoxification of free haem liberated upon host blood digestion is a special challenge for ticks. Thus, it was suggested that GST in the tick midgut might function as an intracellular buffer of labile haem pool to ameliorate its cytotoxic effects upon intracellular hydrolysis of haemoglobin [61]. The authors showed by RNA-seq analysis of *I. ricinus* midguts that expression of this protein was upregulated in the presence of red blood cells in the diet. Interestingly, GST was also more abundant in our study. Additionally, HSP20, accumulated due to rickettsial infection, might be associated with the tick cell response to blood-feeding stress, pathogen infection and questing behaviour [62]. Similarly to myosin-like proteins, HSP20 has been shown to have immunogenic properties in saliva [63]. In our study, putative myosin regulatory light chain and putative myosin class I heavy chain were discovered to be regulated differently in infected *D. reticulatus*. Myosin is a motor protein, best known for its role in ATP-dependent muscle contraction. Non-muscle myosin was reported as highly abundant in the saliva of *I. scapularis* ticks and has been associated with different aspects of wound healing [64]. Increased abundance of two myosin subunits has even been detected in ovaries of *R. microplus* infected with *Babesia*, and for this reason, the molecules have been proposed to be important in successful pathogen transmission [65]. Furthermore, a putative ML-domain protein that was more abundant in response to rickettsial infection in our study might be involved in pathogen recognition and immune response. The gene encoding this protein in *I. ricinus* was found among the genes that are strongly induced by blood meals [66].

Finally, we identified the mitochondrial subunit of ATP synthase, ubiquinone oxidoreductase, and phosphoglycerate kinase, which was accumulated in response to *R. slovaca* infection. These enzymes are essential for glycolysis and electron transport. The ATP synthase was found to be upregulated in the midgut of *Babesia bovis*-infected *R. microplus* ticks [65]. In this study, we also discovered the histone H4 to be more abundant. On the other hand, an ubiquitin-conjugating enzyme E2 and a putative eukaryotic translation initiation factor 4 γ were found to be less abundant in infected ticks. These proteins play a vital role in protein degradation, protein biosynthesis, DNA replication or chromosomal stability. It has been shown that histone H2B has a key role in mediating *Rickettsia felis* internalisation into a tick cell line. It directly interacts with outer-membrane protein B (OmpB), a major rickettsial adhesin molecule. When this protein was depleted, the rickettsial infection in the ISE6 cell line was reduced [67].

## Conclusions

**Fig. 6:**
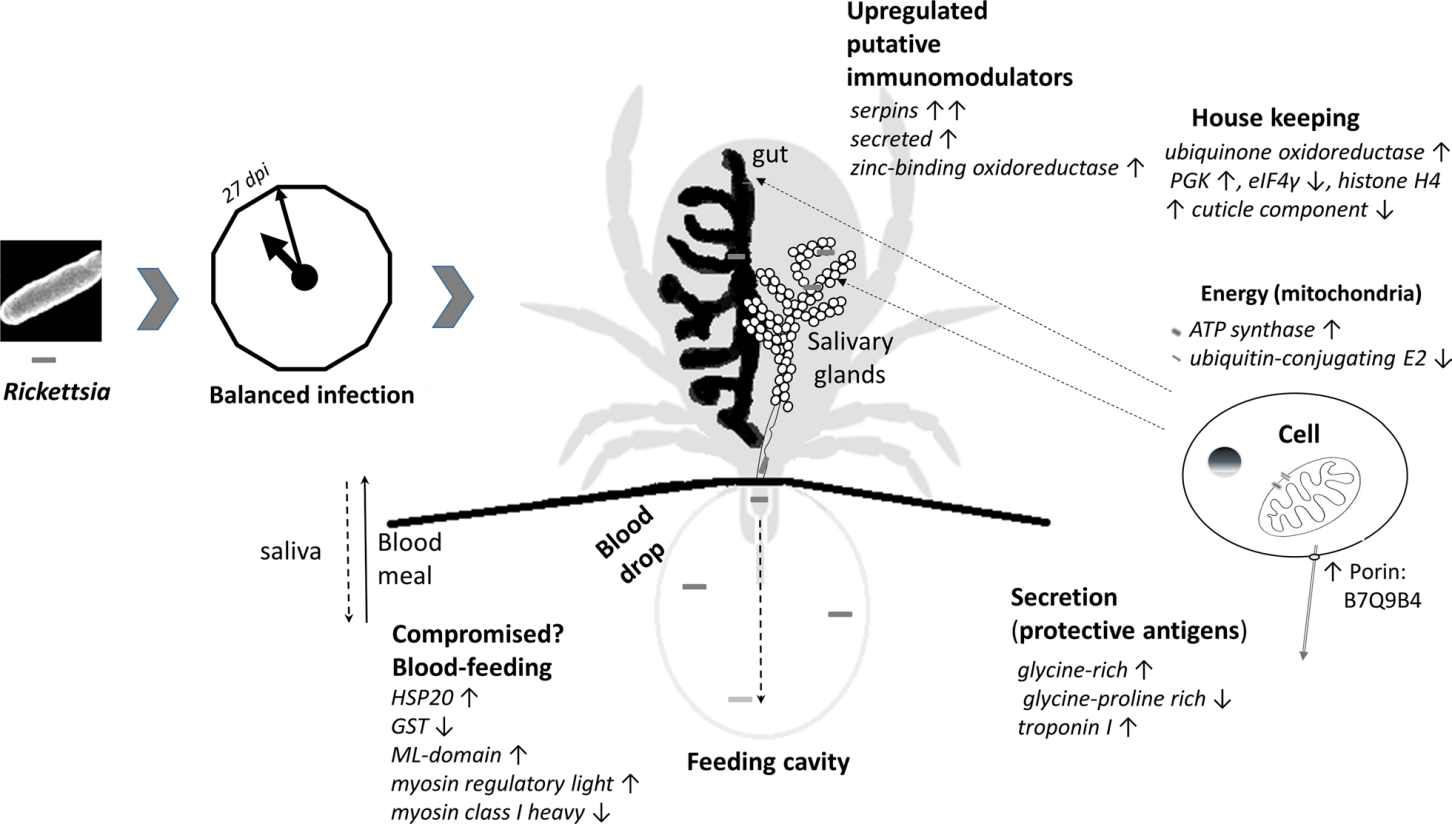
Summary of proteome-wide changes in *Dermacentor reticulatus* induced by capillary tube feeding of the medium containing *Rickettsia slovaca*. *Abbreviations*: GST, glutathione S-transferase; HSP20, heat-shock protein; PGK, phosphoglycerate kinase; eIF, eukaryotic translation initiation factor

This initial comparative proteomic analysis based on 2-DE of the *Dermacentor reticulatus* vector infected with a pathogenic bacterium *Rickettsia slovaca* has revealed 33 differentially abundant proteins in the samples isolated at 27 dpi. The majority have been previously described in other studies in the context of feeding, bacterial infection or defence reaction of the tick (Fig. 6). Some proteins have even been proposed as potential protective antigens or putative immune modulators. Among the proteins discovered as more abundant in infected ticks, we found an uncharacterised protein (UniProt accession B7Q9B4) that may represent a new target for further functional validation. This protein has been bioinformatically predicted to have membrane localisation and porin domain, plausibly playing an important role in the tick-host-pathogen interaction. Considering the functional role of identified differentially abundant proteins, we hypothesise that bacterial infection induces changes that may increase the efficacy of pathogen transmission to the vertebrate host using the tick vector. Thus, this newly acquired knowledge contributes greatly to the clarification of the nature of the vector-pathogen interaction and opens the door to further experiments in the future.

## Additional files

**Additional file 1: Figure S1.** All analytical quality gels included in the comparative proteomic analysis. *Abbreviations*: C, control set; I, infected set.

**Additional file 2: Table S1.** Quantitative data for all gel spots. Statistical test and ratio are shown (positive value spot is more abundant in gels from infected samples, negative value is less abundant). *Abbreviations*: C, control; I, infected; SD, standard deviation.

## Data Availability

The mass spectrometry proteomics data have been deposited to the ProteomeXchange Consortium *via* PRIDE partner repository with dataset identifier PXD011442.

## Abbreviations

2-DE: two-dimensional gel electrophoresis
ABC: ammonium bicarbonate
ACN: acetonitrile
dpi: days post-infection
LC-MS/MS: liquid chromatography coupled to tandem mass spectrometry
GRP: secreted glycine-rich protein
GST: glutathione S-transferase
HSP20: heat-shock protein 20
qPCR: quantitative PCR
RT: room temperature
SDS-PAGE: sodium dodecyl sulfate-polyacrylamide gel electrophoresis
TIBOLA: tick-borne lymphadenopathy
