# Supplementary figures and images for: Comparative proteomics of the vector Dermacentor reticulatus revealed differentially regulated proteins associated with pathogen transmission in response to laboratory infection with Rickettsia slovaca
Source: Parasit Vectors. 2019 Jun 24;12:318. doi: 10.1186/s13071-019-3564-y (PMC6591964; doi:10.1186/s13071-019-3564-y)

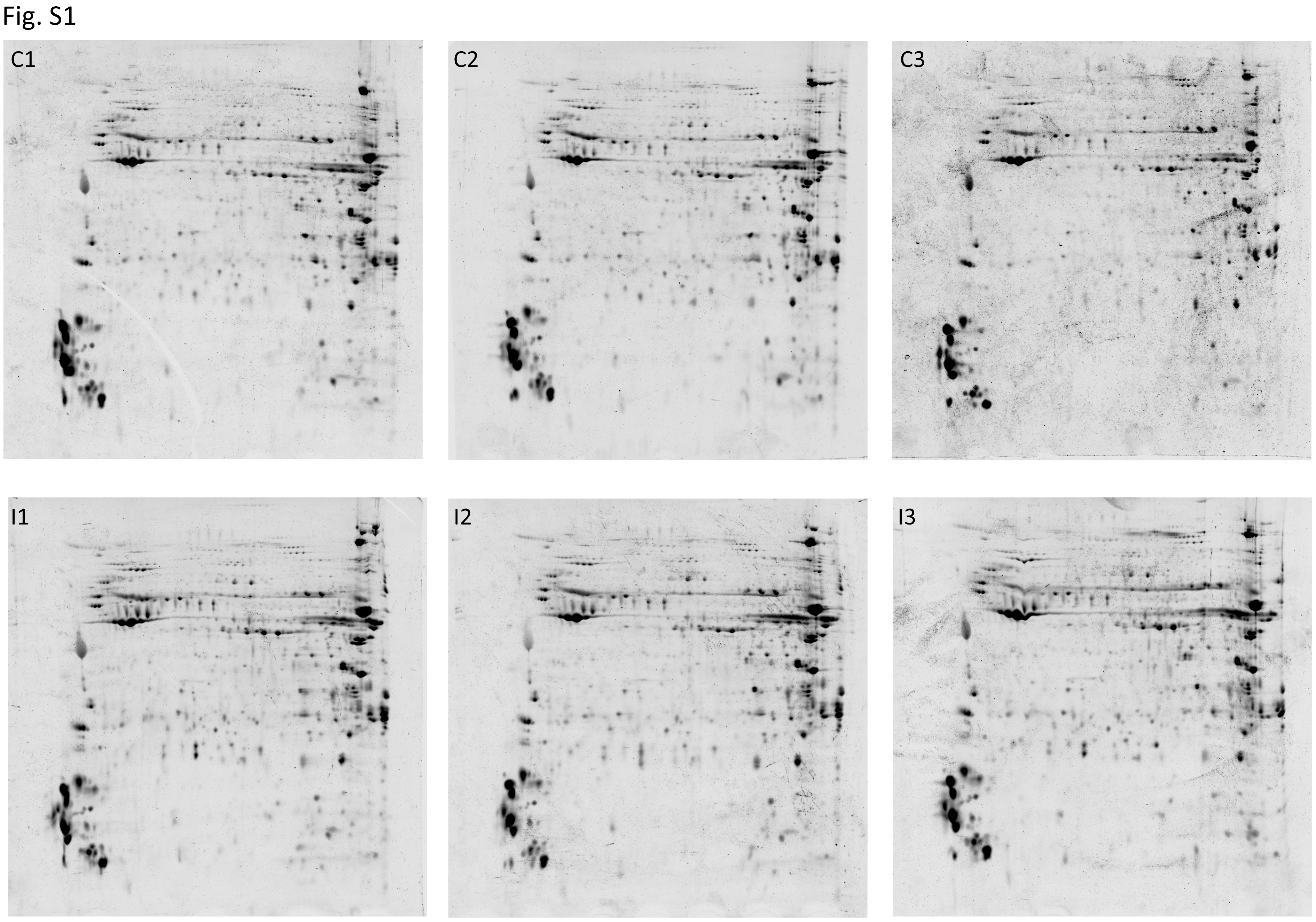

Supplement: Supplementary file 1 — Additional file 1: Figure S1. All analytical quality gels included in the comparative proteomic analysis. Abbreviations: C, control set; I, infected set. [file 13071_2019_3564_MOESM1_ESM.tif]
