# Supplementary material for: Comparative proteomics of the vector Dermacentor reticulatus revealed differentially regulated proteins associated with pathogen transmission in response to laboratory infection with Rickettsia slovaca
Source: Parasit Vectors. 2019 Jun 24;12:318. doi: 10.1186/s13071-019-3564-y (PMC6591964; doi:10.1186/s13071-019-3564-y)
Supplement: Supplementary file 2 — Additional file 2: Table S1. Quantitative data for all gel spots. Statistical test and ratio are shown (positive value spot is more abundant in gels from infected samples, negative value is less abundant). Abbreviations: C, control; I, infected; SD, standard deviation. [file 13071_2019_3564_MOESM2_ESM.docx]

**Additional file 2: Table S1.** **Quantitative data for all gel spots**. Statistical test and ratio are shown (positive value spot is more abundant in gels from infected samples, negative value—less abundant). Abbreviations: C—control, I—infected, SD—standard deviation.

| **Spot** | **Anova (p)** | **Fold** | **Normalized Volume** | | | | | | | | | |
| --- | --- | --- | --- | --- | --- | --- | --- | --- | --- | --- | --- | --- |
|  |  |  | **C1** | **C2** | **C3** | **C-Average** | **C-SD** | **I1** | **I2** | **I3** | **I-Average** | **I-SD** |
| 1754 | 0.0000 | -7.47 | 3.88E+05 | 3.64E+05 | 3.11E+05 | 3.55E+05 | 3.92E+04 | 4.75E+04 | 5.01E+04 | 4.47E+04 | 4.75E+04 | 2.68E+03 |
| 3007 | 0.0000 | 4.35 | 4.50E+04 | 4.85E+04 | 4.25E+04 | 4.53E+04 | 2.98E+03 | 2.07E+05 | 2.04E+05 | 1.81E+05 | 1.97E+05 | 1.39E+04 |
| 2791 | 0.0002 | 2.07 | 2.14E+05 | 2.14E+05 | 2.01E+05 | 2.10E+05 | 7.17E+03 | 4.64E+05 | 3.94E+05 | 4.42E+05 | 4.34E+05 | 3.56E+04 |
| 2923 | 0.0002 | 1.83 | 6.27E+04 | 7.05E+04 | 6.06E+04 | 6.46E+04 | 5.21E+03 | 1.16E+05 | 1.18E+05 | 1.20E+05 | 1.18E+05 | 2.06E+03 |
| 1897 | 0.0010 | 6.09 | 1.28E+05 | 7.22E+04 | 9.41E+04 | 9.80E+04 | 2.80E+04 | 7.58E+05 | 5.38E+05 | 4.95E+05 | 5.97E+05 | 1.41E+05 |
| 3910 | 0.0011 | -3.30 | 6.76E+05 | 6.07E+05 | 6.14E+05 | 6.32E+05 | 3.78E+04 | 2.09E+05 | 2.24E+05 | 1.42E+05 | 1.92E+05 | 4.37E+04 |
| 2833 | 0.0016 | 5.61 | 7.02E+04 | 3.59E+04 | 7.05E+04 | 5.88E+04 | 1.99E+04 | 3.72E+05 | 2.97E+05 | 3.21E+05 | 3.30E+05 | 3.83E+04 |
| 2129 | 0.0023 | 2.22 | 4.41E+04 | 4.96E+04 | 3.95E+04 | 4.44E+04 | 5.03E+03 | 1.18E+05 | 8.87E+04 | 8.91E+04 | 9.87E+04 | 1.69E+04 |
| 1489 | 0.0029 | 3.81 | 4.31E+04 | 6.70E+04 | 8.25E+04 | 6.42E+04 | 1.98E+04 | 2.82E+05 | 2.44E+05 | 2.08E+05 | 2.44E+05 | 3.71E+04 |
| 2740 | 0.0033 | -4.85 | 1.80E+05 | 2.12E+05 | 1.15E+05 | 1.69E+05 | 4.95E+04 | 3.85E+04 | 2.41E+04 | 4.20E+04 | 3.48E+04 | 9.50E+03 |
| 3035 | 0.0036 | 2.35 | 7.07E+04 | 7.20E+04 | 1.06E+05 | 8.28E+04 | 1.98E+04 | 1.79E+05 | 1.90E+05 | 2.15E+05 | 1.95E+05 | 1.83E+04 |
| 3025 | 0.0043 | 7.09 | 7.71E+04 | 1.04E+05 | 1.26E+05 | 1.03E+05 | 2.45E+04 | 1.10E+06 | 4.00E+05 | 6.79E+05 | 7.27E+05 | 3.54E+05 |
| 2653 | 0.0051 | 1.86 | 8.36E+04 | 8.41E+04 | 9.90E+04 | 8.89E+04 | 8.77E+03 | 1.98E+05 | 1.52E+05 | 1.46E+05 | 1.66E+05 | 2.84E+04 |
| 2768 | 0.0052 | -1.54 | 3.59E+06 | 3.41E+06 | 2.82E+06 | 3.27E+06 | 4.02E+05 | 2.15E+06 | 2.19E+06 | 2.02E+06 | 2.12E+06 | 9.18E+04 |
| 1482 | 0.0057 | 1.73 | 8.73E+05 | 9.63E+05 | 8.82E+05 | 9.06E+05 | 4.94E+04 | 1.88E+06 | 1.38E+06 | 1.45E+06 | 1.57E+06 | 2.71E+05 |
| 3596 | 0.0067 | 2.61 | 3.78E+04 | 4.56E+04 | 6.65E+04 | 5.00E+04 | 1.49E+04 | 1.14E+05 | 1.55E+05 | 1.22E+05 | 1.30E+05 | 2.13E+04 |
| 2947 | 0.0080 | 1.89 | 1.92E+05 | 1.86E+05 | 1.45E+05 | 1.75E+05 | 2.57E+04 | 3.19E+05 | 3.89E+05 | 2.82E+05 | 3.30E+05 | 5.46E+04 |
| 1602 | 0.0082 | 1.72 | 2.96E+05 | 4.00E+05 | 3.06E+05 | 3.34E+05 | 5.73E+04 | 6.19E+05 | 5.92E+05 | 5.09E+05 | 5.74E+05 | 5.75E+04 |
| 3421 | 0.0097 | 2.08 | 7.94E+04 | 5.36E+04 | 7.69E+04 | 6.99E+04 | 1.42E+04 | 1.24E+05 | 1.72E+05 | 1.39E+05 | 1.45E+05 | 2.47E+04 |
| 1976 | 0.0164 | 1.53 | 1.74E+05 | 1.35E+05 | 1.34E+05 | 1.47E+05 | 2.29E+04 | 2.11E+05 | 2.54E+05 | 2.09E+05 | 2.25E+05 | 2.55E+04 |
| 2707 | 0.0176 | 3.07 | 5.32E+05 | 5.37E+05 | 2.67E+05 | 4.45E+05 | 1.54E+05 | 1.36E+06 | 1.78E+06 | 9.58E+05 | 1.37E+06 | 4.10E+05 |
| 2818 | 0.0201 | -1.63 | 3.63E+04 | 3.33E+04 | 3.45E+04 | 3.47E+04 | 1.56E+03 | 1.97E+04 | 1.73E+04 | 2.70E+04 | 2.13E+04 | 5.04E+03 |
| 305 | 0.0202 | -1.54 | 6.41E+04 | 7.45E+04 | 9.00E+04 | 7.62E+04 | 1.30E+04 | 4.55E+04 | 4.84E+04 | 5.50E+04 | 4.97E+04 | 4.89E+03 |
| 1808 | 0.0230 | -4.01 | 3.99E+05 | 3.60E+05 | 2.45E+05 | 3.35E+05 | 8.04E+04 | 1.02E+05 | 1.15E+05 | 3.38E+04 | 8.35E+04 | 4.35E+04 |
| 5046 | 0.0258 | -1.74 | 3.92E+06 | 3.81E+06 | 2.78E+06 | 3.50E+06 | 6.28E+05 | 2.17E+06 | 1.58E+06 | 2.30E+06 | 2.02E+06 | 3.83E+05 |
| 1945 | 0.0323 | 1.63 | 7.61E+05 | 7.98E+05 | 4.87E+05 | 6.82E+05 | 1.70E+05 | 1.11E+06 | 1.14E+06 | 1.08E+06 | 1.11E+06 | 2.65E+04 |
| 4628 | 0.0341 | 5.78 | 2.63E+04 | 2.12E+04 | 1.96E+04 | 2.24E+04 | 3.52E+03 | 1.82E+05 | 1.65E+05 | 4.02E+04 | 1.29E+05 | 7.75E+04 |
| 5154 | 0.0370 | -1.76 | 3.06E+05 | 3.86E+05 | 3.28E+05 | 3.40E+05 | 4.11E+04 | 2.61E+05 | 1.79E+05 | 1.41E+05 | 1.93E+05 | 6.12E+04 |
| 2715 | 0.0374 | 2.13 | 9.48E+04 | 1.11E+05 | 5.47E+04 | 8.68E+04 | 2.90E+04 | 2.37E+05 | 1.68E+05 | 1.50E+05 | 1.85E+05 | 4.57E+04 |
| 2917 | 0.0377 | 1.85 | 2.96E+05 | 3.59E+05 | 2.60E+05 | 3.05E+05 | 5.02E+04 | 4.63E+05 | 7.67E+05 | 4.61E+05 | 5.63E+05 | 1.76E+05 |
| 1601 | 0.0431 | 1.83 | 6.75E+05 | 5.02E+05 | 5.13E+05 | 5.63E+05 | 9.69E+04 | 7.24E+05 | 1.04E+06 | 1.32E+06 | 1.03E+06 | 2.98E+05 |
| 3103 | 0.0473 | 1.51 | 2.33E+05 | 2.60E+05 | 1.62E+05 | 2.18E+05 | 5.08E+04 | 3.55E+05 | 3.00E+05 | 3.32E+05 | 3.29E+05 | 2.76E+04 |
| 3871 | 0.0482 | -3.29 | 8.58E+04 | 1.36E+05 | 6.21E+04 | 9.46E+04 | 3.78E+04 | 1.21E+04 | 4.72E+04 | 2.69E+04 | 2.87E+04 | 1.77E+04 |
| 2731 | 0.0030 | 1.34 | 9.38E+05 | 8.59E+05 | 9.35E+05 | 9.11E+05 | 4.48E+04 | 1.31E+06 | 1.19E+06 | 1.16E+06 | 1.22E+06 | 7.57E+04 |
| 5049 | 0.0050 | -1.33 | 6.29E+05 | 6.73E+05 | 6.81E+05 | 6.61E+05 | 2.78E+04 | 5.40E+05 | 4.64E+05 | 4.91E+05 | 4.99E+05 | 3.85E+04 |
| 2802 | 0.0173 | -1.32 | 5.32E+05 | 4.85E+05 | 5.03E+05 | 5.07E+05 | 2.35E+04 | 3.38E+05 | 3.96E+05 | 4.22E+05 | 3.85E+05 | 4.28E+04 |
| 311 | 0.0185 | -1.45 | 1.85E+05 | 2.48E+05 | 2.25E+05 | 2.19E+05 | 3.21E+04 | 1.40E+05 | 1.57E+05 | 1.57E+05 | 1.51E+05 | 1.01E+04 |
| 3514 | 0.0197 | -1.35 | 9.41E+06 | 1.02E+07 | 7.90E+06 | 9.17E+06 | 1.16E+06 | 7.06E+06 | 6.77E+06 | 6.54E+06 | 6.79E+06 | 2.60E+05 |
| 5072 | 0.0215 | -1.27 | 1.81E+05 | 2.11E+05 | 2.21E+05 | 2.04E+05 | 2.07E+04 | 1.67E+05 | 1.53E+05 | 1.61E+05 | 1.61E+05 | 6.94E+03 |
| 2526 | 0.0221 | 1.31 | 1.34E+06 | 1.47E+06 | 1.27E+06 | 1.36E+06 | 1.00E+05 | 1.97E+06 | 1.75E+06 | 1.61E+06 | 1.78E+06 | 1.84E+05 |
| 1703 | 0.0253 | -1.35 | 4.30E+05 | 4.67E+05 | 4.20E+05 | 4.39E+05 | 2.47E+04 | 3.30E+05 | 2.76E+05 | 3.67E+05 | 3.24E+05 | 4.55E+04 |
| 1386 | 0.0258 | 1.35 | 1.67E+05 | 2.01E+05 | 1.61E+05 | 1.76E+05 | 2.17E+04 | 2.57E+05 | 2.42E+05 | 2.14E+05 | 2.38E+05 | 2.16E+04 |
| 3979 | 0.0322 | -1.41 | 1.93E+07 | 1.78E+07 | 1.37E+07 | 1.69E+07 | 2.88E+06 | 1.22E+07 | 1.20E+07 | 1.18E+07 | 1.20E+07 | 1.99E+05 |
| 5059 | 0.0350 | 1.45 | 1.49E+05 | 1.34E+05 | 1.26E+05 | 1.36E+05 | 1.18E+04 | 2.21E+05 | 1.59E+05 | 2.14E+05 | 1.98E+05 | 3.40E+04 |
| 2574 | 0.0361 | -1.47 | 7.97E+04 | 6.02E+04 | 7.25E+04 | 7.08E+04 | 9.88E+03 | 5.39E+04 | 5.08E+04 | 3.98E+04 | 4.82E+04 | 7.46E+03 |
| 488 | 0.0366 | -1.30 | 1.09E+05 | 1.20E+05 | 9.82E+04 | 1.09E+05 | 1.08E+04 | 9.09E+04 | 8.70E+04 | 7.43E+04 | 8.40E+04 | 8.68E+03 |
| 2948 | 0.0376 | 1.48 | 9.94E+04 | 1.17E+05 | 7.71E+04 | 9.79E+04 | 2.01E+04 | 1.46E+05 | 1.58E+05 | 1.32E+05 | 1.45E+05 | 1.32E+04 |
| 506 | 0.0443 | 1.47 | 4.96E+04 | 6.71E+04 | 6.02E+04 | 5.90E+04 | 8.79E+03 | 7.23E+04 | 8.58E+04 | 1.02E+05 | 8.66E+04 | 1.46E+04 |
| 924 | 0.0512 | -1.82 | 9.47E+04 | 1.08E+05 | 1.08E+05 | 1.03E+05 | 7.55E+03 | 5.62E+04 | 7.87E+04 | 3.53E+04 | 5.67E+04 | 2.17E+04 |
| 1587 | 0.0521 | -1.31 | 4.60E+06 | 4.75E+06 | 3.54E+06 | 4.30E+06 | 6.57E+05 | 3.39E+06 | 3.14E+06 | 3.33E+06 | 3.28E+06 | 1.28E+05 |
| 872 | 0.0534 | -1.88 | 4.43E+04 | 4.42E+04 | 4.92E+04 | 4.59E+04 | 2.82E+03 | 2.27E+04 | 3.57E+04 | 1.48E+04 | 2.44E+04 | 1.05E+04 |
| 372 | 0.0542 | 1.25 | 8.25E+04 | 8.99E+04 | 6.81E+04 | 8.02E+04 | 1.11E+04 | 1.05E+05 | 1.01E+05 | 9.61E+04 | 1.01E+05 | 4.34E+03 |
| 1205 | 0.0549 | -1.45 | 2.00E+05 | 1.98E+05 | 1.68E+05 | 1.89E+05 | 1.79E+04 | 1.17E+05 | 1.66E+05 | 1.07E+05 | 1.30E+05 | 3.15E+04 |
| 1620 | 0.0567 | 1.62 | 1.51E+06 | 1.73E+06 | 9.44E+05 | 1.39E+06 | 4.06E+05 | 2.00E+06 | 2.38E+06 | 2.41E+06 | 2.26E+06 | 2.28E+05 |
| 1641 | 0.0578 | 2.72 | 6.01E+05 | 6.16E+05 | 2.02E+05 | 4.73E+05 | 2.35E+05 | 1.76E+06 | 1.16E+06 | 9.44E+05 | 1.29E+06 | 4.21E+05 |
| 1812 | 0.0583 | -1.31 | 5.07E+05 | 4.84E+05 | 4.79E+05 | 4.90E+05 | 1.51E+04 | 3.79E+05 | 4.38E+05 | 3.04E+05 | 3.73E+05 | 6.70E+04 |
| 1463 | 0.0585 | 1.50 | 1.04E+06 | 1.07E+06 | 7.50E+05 | 9.55E+05 | 1.78E+05 | 1.71E+06 | 1.42E+06 | 1.19E+06 | 1.44E+06 | 2.63E+05 |
| 2941 | 0.0585 | 2.46 | 6.12E+04 | 1.73E+05 | 1.08E+05 | 1.14E+05 | 5.61E+04 | 1.85E+05 | 3.65E+05 | 2.93E+05 | 2.81E+05 | 9.08E+04 |
| 2151 | 0.0590 | 1.22 | 1.07E+05 | 8.74E+04 | 8.73E+04 | 9.37E+04 | 1.11E+04 | 1.10E+05 | 1.10E+05 | 1.24E+05 | 1.14E+05 | 8.09E+03 |
| 2771 | 0.0592 | 2.18 | 1.12E+05 | 1.57E+05 | 7.74E+04 | 1.15E+05 | 3.97E+04 | 3.12E+05 | 1.56E+05 | 2.87E+05 | 2.52E+05 | 8.36E+04 |
| 5064 | 0.0605 | 1.47 | 2.22E+05 | 2.30E+05 | 1.50E+05 | 2.01E+05 | 4.40E+04 | 3.15E+05 | 3.12E+05 | 2.56E+05 | 2.94E+05 | 3.33E+04 |
| 1691 | 0.0616 | -1.59 | 4.24E+05 | 4.21E+05 | 2.56E+05 | 3.67E+05 | 9.62E+04 | 2.21E+05 | 2.44E+05 | 2.28E+05 | 2.31E+05 | 1.19E+04 |
| 4250 | 0.0671 | -1.62 | 8.92E+04 | 1.11E+05 | 6.96E+04 | 8.99E+04 | 2.07E+04 | 5.65E+04 | 4.20E+04 | 6.79E+04 | 5.55E+04 | 1.30E+04 |
| 4066 | 0.0700 | -1.41 | 8.79E+06 | 7.08E+06 | 5.62E+06 | 7.16E+06 | 1.58E+06 | 5.28E+06 | 4.67E+06 | 5.25E+06 | 5.07E+06 | 3.43E+05 |
| 5127 | 0.0719 | -1.21 | 8.10E+05 | 1.02E+06 | 8.32E+05 | 8.87E+05 | 1.14E+05 | 7.51E+05 | 7.01E+05 | 7.50E+05 | 7.34E+05 | 2.90E+04 |
| 1745 | 0.0725 | 1.54 | 1.38E+04 | 1.98E+04 | 1.21E+04 | 1.52E+04 | 4.04E+03 | 2.69E+04 | 1.88E+04 | 2.46E+04 | 2.34E+04 | 4.20E+03 |
| 1619 | 0.0738 | 1.46 | 4.33E+05 | 4.66E+05 | 2.78E+05 | 3.92E+05 | 1.01E+05 | 5.41E+05 | 5.57E+05 | 6.23E+05 | 5.74E+05 | 4.38E+04 |
| 5047 | 0.0750 | 1.58 | 6.43E+04 | 8.70E+04 | 7.14E+04 | 7.42E+04 | 1.16E+04 | 1.26E+05 | 1.43E+05 | 8.35E+04 | 1.18E+05 | 3.06E+04 |
| 660 | 0.0758 | 2.57 | 1.93E+04 | 1.56E+04 | 1.73E+04 | 1.74E+04 | 1.87E+03 | 2.26E+04 | 7.36E+04 | 3.77E+04 | 4.46E+04 | 2.62E+04 |
| 4228 | 0.0793 | 1.71 | 2.56E+04 | 1.59E+04 | 3.84E+04 | 2.66E+04 | 1.13E+04 | 4.62E+04 | 4.38E+04 | 4.67E+04 | 4.56E+04 | 1.54E+03 |
| 3430 | 0.0818 | 1.41 | 2.17E+06 | 2.00E+06 | 1.30E+06 | 1.82E+06 | 4.61E+05 | 2.60E+06 | 2.48E+06 | 2.65E+06 | 2.58E+06 | 8.77E+04 |
| 366 | 0.0826 | 1.59 | 3.93E+04 | 3.48E+04 | 1.97E+04 | 3.13E+04 | 1.02E+04 | 4.89E+04 | 5.40E+04 | 4.58E+04 | 4.96E+04 | 4.12E+03 |
| 3117 | 0.0903 | 1.81 | 6.60E+05 | 7.89E+05 | 4.10E+05 | 6.20E+05 | 1.93E+05 | 1.51E+06 | 7.99E+05 | 1.06E+06 | 1.12E+06 | 3.60E+05 |
| 1618 | 0.0908 | 1.82 | 1.74E+05 | 1.67E+05 | 6.96E+04 | 1.37E+05 | 5.84E+04 | 2.28E+05 | 2.44E+05 | 2.74E+05 | 2.49E+05 | 2.36E+04 |
| 1729 | 0.0909 | 1.51 | 2.17E+04 | 2.82E+04 | 1.66E+04 | 2.22E+04 | 5.81E+03 | 4.01E+04 | 2.72E+04 | 3.28E+04 | 3.34E+04 | 6.46E+03 |
| 714 | 0.0933 | -1.27 | 9.27E+04 | 7.43E+04 | 7.87E+04 | 8.19E+04 | 9.64E+03 | 7.38E+04 | 6.54E+04 | 5.47E+04 | 6.46E+04 | 9.55E+03 |
| 1210 | 0.0933 | 2.37 | 3.70E+05 | 3.95E+05 | 1.45E+05 | 3.03E+05 | 1.37E+05 | 5.36E+05 | 5.18E+05 | 1.10E+06 | 7.18E+05 | 3.31E+05 |
| 1927 | 0.0944 | 1.54 | 6.22E+05 | 6.72E+05 | 3.62E+05 | 5.52E+05 | 1.67E+05 | 7.44E+05 | 9.86E+05 | 8.24E+05 | 8.51E+05 | 1.23E+05 |
| 935 | 0.0954 | -1.60 | 3.59E+05 | 3.87E+05 | 2.12E+05 | 3.19E+05 | 9.43E+04 | 2.23E+05 | 1.77E+05 | 2.00E+05 | 2.00E+05 | 2.31E+04 |
| 954 | 0.0963 | 1.39 | 1.19E+05 | 1.07E+05 | 9.29E+04 | 1.06E+05 | 1.29E+04 | 1.45E+05 | 1.83E+05 | 1.17E+05 | 1.48E+05 | 3.32E+04 |
| 1003 | 0.0964 | 1.28 | 1.10E+05 | 1.32E+05 | 8.81E+04 | 1.10E+05 | 2.21E+04 | 1.43E+05 | 1.46E+05 | 1.34E+05 | 1.41E+05 | 6.40E+03 |
| 5061 | 0.0968 | 2.21 | 9.81E+04 | 1.03E+05 | 8.38E+04 | 9.50E+04 | 1.00E+04 | 2.91E+05 | 1.02E+05 | 2.36E+05 | 2.10E+05 | 9.73E+04 |
| 654 | 0.0992 | 2.17 | 8.61E+04 | 4.49E+04 | 5.82E+04 | 6.31E+04 | 2.10E+04 | 1.00E+05 | 2.20E+05 | 9.00E+04 | 1.37E+05 | 7.25E+04 |
| 2858 | 0.1009 | 2.17 | 1.20E+04 | 1.50E+04 | 3.62E+04 | 2.11E+04 | 1.32E+04 | 4.69E+04 | 2.92E+04 | 6.09E+04 | 4.57E+04 | 1.59E+04 |
| 925 | 0.1061 | -1.42 | 1.03E+05 | 1.19E+05 | 1.26E+05 | 1.16E+05 | 1.21E+04 | 7.69E+04 | 1.08E+05 | 6.04E+04 | 8.18E+04 | 2.43E+04 |
| 1305 | 0.1067 | -1.14 | 1.55E+06 | 1.76E+06 | 1.43E+06 | 1.58E+06 | 1.67E+05 | 1.40E+06 | 1.41E+06 | 1.35E+06 | 1.38E+06 | 3.36E+04 |
| 1382 | 0.1075 | 1.61 | 2.70E+04 | 2.26E+04 | 2.64E+04 | 2.53E+04 | 2.39E+03 | 4.49E+04 | 2.62E+04 | 5.12E+04 | 4.07E+04 | 1.30E+04 |
| 2457 | 0.1090 | 1.29 | 9.35E+04 | 9.09E+04 | 6.69E+04 | 8.38E+04 | 1.46E+04 | 1.23E+05 | 9.99E+04 | 1.01E+05 | 1.08E+05 | 1.30E+04 |
| 2191 | 0.1093 | 1.21 | 3.96E+05 | 4.24E+05 | 4.82E+05 | 4.34E+05 | 4.38E+04 | 5.52E+05 | 5.74E+05 | 4.53E+05 | 5.26E+05 | 6.42E+04 |
| 2935 | 0.1101 | -1.52 | 2.76E+05 | 3.17E+05 | 2.41E+05 | 2.78E+05 | 3.76E+04 | 2.12E+05 | 2.21E+05 | 1.17E+05 | 1.83E+05 | 5.77E+04 |
| 1630 | 0.1102 | 1.31 | 1.72E+05 | 1.65E+05 | 1.26E+05 | 1.54E+05 | 2.53E+04 | 2.26E+05 | 1.69E+05 | 2.12E+05 | 2.02E+05 | 2.99E+04 |
| 1614 | 0.1126 | 2.50 | 4.42E+05 | 5.38E+05 | 1.25E+05 | 3.68E+05 | 2.16E+05 | 1.25E+06 | 9.49E+05 | 5.66E+05 | 9.20E+05 | 3.41E+05 |
| 514 | 0.1134 | 1.26 | 4.59E+04 | 6.20E+04 | 5.17E+04 | 5.32E+04 | 8.20E+03 | 5.97E+04 | 6.45E+04 | 7.68E+04 | 6.70E+04 | 8.81E+03 |
| 1396 | 0.1146 | 1.15 | 6.22E+05 | 6.44E+05 | 5.16E+05 | 5.94E+05 | 6.87E+04 | 7.05E+05 | 6.94E+05 | 6.55E+05 | 6.84E+05 | 2.62E+04 |
| 5108 | 0.1154 | 1.40 | 2.12E+05 | 2.04E+05 | 1.73E+05 | 1.96E+05 | 2.04E+04 | 2.81E+05 | 2.05E+05 | 3.36E+05 | 2.74E+05 | 6.62E+04 |
| 5115 | 0.1158 | -1.30 | 5.01E+05 | 4.86E+05 | 3.70E+05 | 4.52E+05 | 7.18E+04 | 2.92E+05 | 3.74E+05 | 3.80E+05 | 3.48E+05 | 4.93E+04 |
| 2728 | 0.1161 | 1.34 | 4.35E+05 | 4.88E+05 | 3.18E+05 | 4.14E+05 | 8.70E+04 | 5.17E+05 | 6.40E+05 | 5.01E+05 | 5.53E+05 | 7.63E+04 |
| 471 | 0.1163 | -1.48 | 9.48E+04 | 8.66E+04 | 1.30E+05 | 1.04E+05 | 2.32E+04 | 5.10E+04 | 7.06E+04 | 8.84E+04 | 7.00E+04 | 1.87E+04 |
| 930 | 0.1166 | -2.03 | 5.22E+04 | 3.39E+04 | 8.88E+04 | 5.83E+04 | 2.80E+04 | 2.28E+04 | 3.99E+04 | 2.35E+04 | 2.88E+04 | 9.69E+03 |
| 1690 | 0.1194 | -1.43 | 2.06E+06 | 2.04E+06 | 1.50E+06 | 1.87E+06 | 3.17E+05 | 1.32E+06 | 1.63E+06 | 9.47E+05 | 1.30E+06 | 3.43E+05 |
| 1993 | 0.1205 | -1.52 | 2.20E+05 | 1.76E+05 | 1.17E+05 | 1.71E+05 | 5.16E+04 | 1.02E+05 | 1.29E+05 | 1.07E+05 | 1.13E+05 | 1.45E+04 |
| 3049 | 0.1212 | 1.70 | 2.55E+05 | 4.87E+05 | 2.90E+05 | 3.44E+05 | 1.25E+05 | 6.25E+05 | 3.92E+05 | 7.39E+05 | 5.85E+05 | 1.77E+05 |
| 1546 | 0.1217 | -1.19 | 1.71E+06 | 1.70E+06 | 1.35E+06 | 1.59E+06 | 2.09E+05 | 1.42E+06 | 1.29E+06 | 1.30E+06 | 1.34E+06 | 7.19E+04 |
| 625 | 0.1219 | -1.28 | 1.02E+05 | 1.05E+05 | 1.27E+05 | 1.11E+05 | 1.40E+04 | 7.34E+04 | 1.06E+05 | 8.22E+04 | 8.72E+04 | 1.68E+04 |
| 2609 | 0.1235 | -1.55 | 2.10E+06 | 2.23E+06 | 1.21E+06 | 1.85E+06 | 5.55E+05 | 1.16E+06 | 1.38E+06 | 1.04E+06 | 1.19E+06 | 1.72E+05 |
| 5053 | 0.1289 | 1.50 | 1.39E+05 | 2.59E+05 | 1.94E+05 | 1.97E+05 | 6.00E+04 | 3.48E+05 | 2.27E+05 | 3.10E+05 | 2.95E+05 | 6.19E+04 |
| 819 | 0.1290 | -1.30 | 3.00E+05 | 2.81E+05 | 3.79E+05 | 3.20E+05 | 5.18E+04 | 2.00E+05 | 2.67E+05 | 2.75E+05 | 2.47E+05 | 4.14E+04 |
| 1826 | 0.1329 | -1.43 | 4.40E+05 | 4.15E+05 | 2.95E+05 | 3.83E+05 | 7.74E+04 | 2.10E+05 | 3.47E+05 | 2.49E+05 | 2.68E+05 | 7.06E+04 |
| 1237 | 0.1335 | -1.13 | 9.27E+05 | 1.01E+06 | 8.88E+05 | 9.40E+05 | 5.98E+04 | 8.12E+05 | 9.21E+05 | 7.66E+05 | 8.33E+05 | 7.92E+04 |
| 915 | 0.1339 | 1.29 | 4.08E+04 | 3.33E+04 | 2.74E+04 | 3.38E+04 | 6.73E+03 | 3.86E+04 | 4.19E+04 | 5.06E+04 | 4.37E+04 | 6.18E+03 |
| 2776 | 0.1376 | -1.67 | 2.15E+06 | 1.95E+06 | 2.04E+06 | 2.05E+06 | 1.04E+05 | 1.61E+06 | 1.48E+06 | 5.83E+05 | 1.22E+06 | 5.59E+05 |
| 1314 | 0.1407 | 1.47 | 1.61E+05 | 1.01E+05 | 8.81E+04 | 1.17E+05 | 3.89E+04 | 2.10E+05 | 1.37E+05 | 1.67E+05 | 1.71E+05 | 3.65E+04 |
| 2844 | 0.1441 | 1.40 | 1.91E+05 | 2.45E+05 | 1.22E+05 | 1.86E+05 | 6.17E+04 | 2.63E+05 | 2.78E+05 | 2.43E+05 | 2.61E+05 | 1.79E+04 |
| 472 | 0.1450 | -1.31 | 3.80E+04 | 4.18E+04 | 4.38E+04 | 4.12E+04 | 2.96E+03 | 2.25E+04 | 3.44E+04 | 3.73E+04 | 3.14E+04 | 7.83E+03 |
| 1116 | 0.1452 | -1.26 | 1.11E+05 | 1.17E+05 | 8.30E+04 | 1.04E+05 | 1.81E+04 | 7.95E+04 | 9.25E+04 | 7.49E+04 | 8.23E+04 | 9.12E+03 |
| 1115 | 0.1454 | -1.43 | 6.72E+04 | 8.44E+04 | 8.97E+04 | 8.04E+04 | 1.18E+04 | 5.77E+04 | 7.32E+04 | 3.73E+04 | 5.61E+04 | 1.80E+04 |
| 638 | 0.1456 | -1.34 | 2.83E+04 | 1.93E+04 | 2.11E+04 | 2.29E+04 | 4.74E+03 | 1.80E+04 | 1.35E+04 | 1.97E+04 | 1.71E+04 | 3.19E+03 |
| 1397 | 0.1503 | 1.38 | 4.39E+04 | 3.86E+04 | 6.60E+04 | 4.95E+04 | 1.46E+04 | 7.10E+04 | 5.57E+04 | 7.78E+04 | 6.82E+04 | 1.13E+04 |
| 1235 | 0.1547 | 1.52 | 1.78E+05 | 1.36E+05 | 1.20E+05 | 1.45E+05 | 3.01E+04 | 2.23E+05 | 1.48E+05 | 2.90E+05 | 2.21E+05 | 7.09E+04 |
| 2666 | 0.1555 | -1.47 | 3.89E+04 | 2.71E+04 | 2.16E+04 | 2.92E+04 | 8.83E+03 | 1.74E+04 | 2.51E+04 | 1.72E+04 | 1.99E+04 | 4.52E+03 |
| 2221 | 0.1604 | 1.28 | 5.97E+04 | 7.31E+04 | 4.53E+04 | 5.93E+04 | 1.39E+04 | 8.32E+04 | 6.68E+04 | 7.85E+04 | 7.62E+04 | 8.45E+03 |
| 589 | 0.1622 | -1.34 | 2.12E+05 | 1.60E+05 | 2.48E+05 | 2.06E+05 | 4.39E+04 | 1.56E+05 | 1.82E+05 | 1.25E+05 | 1.54E+05 | 2.86E+04 |
| 5050 | 0.1622 | -1.39 | 6.94E+05 | 4.68E+05 | 6.32E+05 | 5.98E+05 | 1.17E+05 | 5.39E+05 | 4.39E+05 | 3.13E+05 | 4.30E+05 | 1.13E+05 |
| 1640 | 0.1637 | 1.62 | 2.73E+05 | 5.76E+05 | 3.01E+05 | 3.83E+05 | 1.67E+05 | 7.73E+05 | 4.21E+05 | 6.63E+05 | 6.19E+05 | 1.80E+05 |
| 3038 | 0.1678 | 1.31 | 1.15E+05 | 1.11E+05 | 1.14E+05 | 1.13E+05 | 2.25E+03 | 1.84E+05 | 1.11E+05 | 1.49E+05 | 1.48E+05 | 3.66E+04 |
| 1790 | 0.1684 | 1.26 | 2.05E+05 | 3.07E+05 | 3.30E+05 | 2.81E+05 | 6.64E+04 | 3.51E+05 | 3.48E+05 | 3.59E+05 | 3.53E+05 | 5.48E+03 |
| 1627 | 0.1786 | 1.95 | 9.83E+05 | 1.23E+06 | 3.77E+05 | 8.62E+05 | 4.37E+05 | 2.10E+06 | 2.00E+06 | 9.48E+05 | 1.68E+06 | 6.38E+05 |
| 5153 | 0.1835 | 1.24 | 1.07E+07 | 1.09E+07 | 8.44E+06 | 1.00E+07 | 1.37E+06 | 1.45E+07 | 1.25E+07 | 1.02E+07 | 1.24E+07 | 2.15E+06 |
| 5077 | 0.1842 | -1.14 | 5.18E+05 | 5.88E+05 | 6.15E+05 | 5.74E+05 | 5.04E+04 | 4.65E+05 | 4.71E+05 | 5.71E+05 | 5.02E+05 | 5.93E+04 |
| 2294 | 0.1844 | 1.58 | 3.30E+04 | 3.28E+04 | 4.84E+04 | 3.80E+04 | 8.97E+03 | 3.90E+04 | 5.53E+04 | 8.62E+04 | 6.01E+04 | 2.40E+04 |
| 243 | 0.1884 | 1.27 | 2.74E+04 | 3.58E+04 | 4.48E+04 | 3.60E+04 | 8.71E+03 | 4.11E+04 | 5.31E+04 | 4.35E+04 | 4.59E+04 | 6.33E+03 |
| 1881 | 0.1906 | -1.33 | 1.66E+06 | 2.14E+06 | 1.22E+06 | 1.67E+06 | 4.60E+05 | 1.29E+06 | 1.28E+06 | 1.21E+06 | 1.26E+06 | 4.42E+04 |
| 443 | 0.1908 | -1.32 | 5.15E+04 | 4.93E+04 | 6.84E+04 | 5.64E+04 | 1.05E+04 | 3.10E+04 | 4.84E+04 | 4.89E+04 | 4.28E+04 | 1.02E+04 |
| 2190 | 0.1910 | -1.19 | 3.66E+05 | 4.13E+05 | 2.90E+05 | 3.56E+05 | 6.20E+04 | 2.94E+05 | 3.05E+05 | 2.99E+05 | 2.99E+05 | 5.94E+03 |
| 1392 | 0.1930 | 1.17 | 5.84E+05 | 6.20E+05 | 4.93E+05 | 5.65E+05 | 6.54E+04 | 7.34E+05 | 6.68E+05 | 5.76E+05 | 6.59E+05 | 7.93E+04 |
| 2944 | 0.1951 | 1.29 | 2.43E+05 | 2.84E+05 | 1.70E+05 | 2.32E+05 | 5.74E+04 | 3.23E+05 | 3.22E+05 | 2.52E+05 | 2.99E+05 | 4.11E+04 |
| 1625 | 0.1961 | 1.46 | 7.13E+04 | 9.57E+04 | 7.45E+04 | 8.05E+04 | 1.32E+04 | 1.17E+05 | 7.81E+04 | 1.58E+05 | 1.18E+05 | 4.01E+04 |
| 1843 | 0.1998 | -1.33 | 5.71E+05 | 7.34E+05 | 4.33E+05 | 5.79E+05 | 1.51E+05 | 4.76E+05 | 3.59E+05 | 4.68E+05 | 4.34E+05 | 6.56E+04 |
| 1941 | 0.2048 | -1.27 | 1.72E+05 | 1.69E+05 | 1.46E+05 | 1.62E+05 | 1.44E+04 | 1.53E+05 | 1.40E+05 | 8.93E+04 | 1.28E+05 | 3.37E+04 |
| 1997 | 0.2062 | -1.19 | 2.30E+05 | 2.02E+05 | 1.64E+05 | 1.99E+05 | 3.30E+04 | 1.76E+05 | 1.75E+05 | 1.49E+05 | 1.67E+05 | 1.53E+04 |
| 2367 | 0.2062 | 1.20 | 4.32E+06 | 4.64E+06 | 3.26E+06 | 4.08E+06 | 7.22E+05 | 5.19E+06 | 5.25E+06 | 4.26E+06 | 4.90E+06 | 5.55E+05 |
| 1742 | 0.2072 | 1.46 | 4.54E+04 | 3.72E+04 | 6.83E+04 | 5.03E+04 | 1.61E+04 | 6.13E+04 | 1.01E+05 | 5.79E+04 | 7.33E+04 | 2.38E+04 |
| 1198 | 0.2073 | -1.12 | 1.77E+05 | 1.71E+05 | 1.41E+05 | 1.63E+05 | 1.92E+04 | 1.50E+05 | 1.43E+05 | 1.43E+05 | 1.45E+05 | 4.07E+03 |
| 640 | 0.2103 | 1.27 | 1.06E+05 | 1.06E+05 | 8.88E+04 | 1.00E+05 | 9.99E+03 | 1.63E+05 | 1.15E+05 | 1.04E+05 | 1.27E+05 | 3.14E+04 |
| 2765 | 0.2115 | -1.12 | 1.93E+06 | 1.76E+06 | 1.61E+06 | 1.77E+06 | 1.58E+05 | 1.74E+06 | 1.56E+06 | 1.45E+06 | 1.58E+06 | 1.48E+05 |
| 1131 | 0.2122 | -1.28 | 5.87E+04 | 4.60E+04 | 3.67E+04 | 4.71E+04 | 1.11E+04 | 4.18E+04 | 3.72E+04 | 3.12E+04 | 3.67E+04 | 5.31E+03 |
| 664 | 0.2162 | -1.32 | 1.02E+05 | 6.82E+04 | 7.46E+04 | 8.16E+04 | 1.81E+04 | 8.15E+04 | 5.27E+04 | 5.17E+04 | 6.20E+04 | 1.69E+04 |
| 1327 | 0.2208 | -1.12 | 6.35E+05 | 7.32E+05 | 5.65E+05 | 6.44E+05 | 8.35E+04 | 5.95E+05 | 5.56E+05 | 5.68E+05 | 5.73E+05 | 2.02E+04 |
| 1043 | 0.2210 | 1.19 | 2.09E+05 | 2.03E+05 | 1.41E+05 | 1.85E+05 | 3.75E+04 | 2.27E+05 | 2.14E+05 | 2.15E+05 | 2.19E+05 | 7.27E+03 |
| 3302 | 0.2245 | 1.22 | 1.42E+05 | 1.48E+05 | 9.47E+04 | 1.28E+05 | 2.92E+04 | 1.72E+05 | 1.50E+05 | 1.48E+05 | 1.56E+05 | 1.34E+04 |
| 1249 | 0.2254 | -1.29 | 6.75E+05 | 7.49E+05 | 5.29E+05 | 6.51E+05 | 1.12E+05 | 5.60E+05 | 5.91E+05 | 3.63E+05 | 5.05E+05 | 1.24E+05 |
| 758 | 0.2257 | -1.73 | 4.49E+04 | 2.27E+04 | 6.44E+04 | 4.40E+04 | 2.09E+04 | 1.90E+04 | 2.84E+04 | 2.88E+04 | 2.54E+04 | 5.56E+03 |
| 1732 | 0.2265 | 1.33 | 3.34E+05 | 3.81E+05 | 1.76E+05 | 2.97E+05 | 1.07E+05 | 4.03E+05 | 3.98E+05 | 3.87E+05 | 3.96E+05 | 8.11E+03 |
| 2001 | 0.2279 | 1.03 | 1.98E+05 | 1.92E+05 | 1.98E+05 | 1.96E+05 | 3.45E+03 | 1.94E+05 | 2.07E+05 | 2.07E+05 | 2.03E+05 | 7.39E+03 |
| 3917 | 0.2300 | -1.56 | 3.95E+05 | 5.47E+05 | 2.32E+05 | 3.91E+05 | 1.58E+05 | 2.73E+05 | 1.88E+05 | 2.91E+05 | 2.51E+05 | 5.50E+04 |
| 5142 | 0.2311 | 1.36 | 4.49E+06 | 5.72E+06 | 3.02E+06 | 4.41E+06 | 1.35E+06 | 7.57E+06 | 5.59E+06 | 4.82E+06 | 5.99E+06 | 1.42E+06 |
| 826 | 0.2312 | 1.32 | 3.25E+05 | 2.83E+05 | 2.50E+05 | 2.86E+05 | 3.76E+04 | 4.46E+05 | 4.20E+05 | 2.65E+05 | 3.77E+05 | 9.80E+04 |
| 2013 | 0.2320 | 1.28 | 9.08E+04 | 1.04E+05 | 1.35E+05 | 1.10E+05 | 2.26E+04 | 1.12E+05 | 1.35E+05 | 1.74E+05 | 1.40E+05 | 3.11E+04 |
| 1846 | 0.2324 | -1.24 | 2.74E+05 | 2.83E+05 | 2.61E+05 | 2.72E+05 | 1.09E+04 | 2.39E+05 | 2.67E+05 | 1.53E+05 | 2.20E+05 | 5.94E+04 |
| 632 | 0.2356 | -1.75 | 3.63E+04 | 1.54E+04 | 4.56E+04 | 3.25E+04 | 1.55E+04 | 1.74E+04 | 2.06E+04 | 1.75E+04 | 1.85E+04 | 1.81E+03 |
| 397 | 0.2396 | -1.64 | 7.68E+04 | 1.21E+05 | 1.21E+05 | 1.06E+05 | 2.56E+04 | 2.31E+04 | 1.14E+05 | 5.70E+04 | 6.48E+04 | 4.60E+04 |
| 2232 | 0.2424 | 1.32 | 7.33E+04 | 1.35E+05 | 7.22E+04 | 9.35E+04 | 3.59E+04 | 1.38E+05 | 1.01E+05 | 1.30E+05 | 1.23E+05 | 1.98E+04 |
| 891 | 0.2426 | -1.29 | 4.11E+05 | 4.32E+05 | 2.85E+05 | 3.76E+05 | 7.95E+04 | 3.43E+05 | 2.25E+05 | 3.10E+05 | 2.93E+05 | 6.09E+04 |
| 624 | 0.2426 | -1.24 | 9.76E+04 | 1.15E+05 | 1.00E+05 | 1.04E+05 | 9.23E+03 | 5.92E+04 | 1.02E+05 | 9.06E+04 | 8.40E+04 | 2.23E+04 |
| 1356 | 0.2452 | 1.08 | 2.27E+05 | 2.26E+05 | 2.29E+05 | 2.27E+05 | 1.52E+03 | 2.64E+05 | 2.56E+05 | 2.20E+05 | 2.46E+05 | 2.36E+04 |
| 2887 | 0.2481 | 1.17 | 4.23E+05 | 4.31E+05 | 3.29E+05 | 3.94E+05 | 5.68E+04 | 5.38E+05 | 4.28E+05 | 4.20E+05 | 4.62E+05 | 6.58E+04 |
| 5060 | 0.2498 | -1.19 | 8.98E+06 | 8.69E+06 | 6.27E+06 | 7.98E+06 | 1.49E+06 | 6.90E+06 | 6.11E+06 | 7.06E+06 | 6.69E+06 | 5.09E+05 |
| 1600 | 0.2525 | 1.42 | 2.52E+05 | 1.84E+05 | 3.37E+05 | 2.58E+05 | 7.69E+04 | 2.43E+05 | 3.99E+05 | 4.54E+05 | 3.65E+05 | 1.09E+05 |
| 731 | 0.2565 | 1.20 | 9.95E+04 | 7.42E+04 | 7.10E+04 | 8.16E+04 | 1.56E+04 | 8.05E+04 | 1.06E+05 | 1.09E+05 | 9.83E+04 | 1.55E+04 |
| 3632 | 0.2585 | -1.29 | 8.65E+06 | 7.14E+06 | 4.86E+06 | 6.88E+06 | 1.91E+06 | 5.75E+06 | 5.29E+06 | 4.95E+06 | 5.33E+06 | 4.05E+05 |
| 641 | 0.2665 | 1.18 | 3.92E+04 | 3.78E+04 | 3.06E+04 | 3.59E+04 | 4.60E+03 | 5.08E+04 | 4.00E+04 | 3.63E+04 | 4.24E+04 | 7.54E+03 |
| 1683 | 0.2667 | -1.17 | 1.27E+06 | 1.38E+06 | 9.61E+05 | 1.20E+06 | 2.17E+05 | 9.43E+05 | 1.07E+06 | 1.06E+06 | 1.03E+06 | 7.19E+04 |
| 2562 | 0.2671 | -1.46 | 1.74E+06 | 1.92E+06 | 9.26E+05 | 1.53E+06 | 5.28E+05 | 8.06E+05 | 1.34E+06 | 1.00E+06 | 1.05E+06 | 2.70E+05 |
| 491 | 0.2689 | -1.15 | 1.01E+05 | 1.09E+05 | 8.65E+04 | 9.91E+04 | 1.16E+04 | 8.80E+04 | 9.78E+04 | 7.26E+04 | 8.61E+04 | 1.27E+04 |
| 2130 | 0.2690 | -1.18 | 6.57E+04 | 5.31E+04 | 4.36E+04 | 5.41E+04 | 1.11E+04 | 4.56E+04 | 4.44E+04 | 4.74E+04 | 4.58E+04 | 1.48E+03 |
| 5135 | 0.2726 | -1.13 | 6.00E+06 | 6.41E+06 | 5.84E+06 | 6.08E+06 | 2.96E+05 | 6.49E+06 | 4.82E+06 | 4.90E+06 | 5.40E+06 | 9.41E+05 |
| 2813 | 0.2812 | 1.32 | 1.73E+05 | 2.62E+05 | 1.93E+05 | 2.10E+05 | 4.68E+04 | 3.54E+05 | 1.95E+05 | 2.83E+05 | 2.77E+05 | 7.96E+04 |
| 1438 | 0.2813 | 1.35 | 4.80E+04 | 6.07E+04 | 2.77E+04 | 4.55E+04 | 1.67E+04 | 5.36E+04 | 5.20E+04 | 7.86E+04 | 6.14E+04 | 1.49E+04 |
| 685 | 0.2821 | 1.31 | 7.08E+04 | 6.14E+04 | 2.94E+04 | 5.39E+04 | 2.17E+04 | 7.13E+04 | 7.22E+04 | 6.87E+04 | 7.07E+04 | 1.81E+03 |
| 2735 | 0.2860 | 1.33 | 3.89E+05 | 4.31E+05 | 1.78E+05 | 3.33E+05 | 1.35E+05 | 4.67E+05 | 4.62E+05 | 3.95E+05 | 4.41E+05 | 3.99E+04 |
| 4354 | 0.2876 | -1.19 | 1.54E+06 | 1.36E+06 | 1.12E+06 | 1.34E+06 | 2.08E+05 | 1.38E+06 | 1.04E+06 | 9.45E+05 | 1.12E+06 | 2.30E+05 |
| 3274 | 0.2879 | 1.11 | 4.24E+05 | 3.89E+05 | 3.70E+05 | 3.94E+05 | 2.76E+04 | 4.67E+05 | 4.65E+05 | 3.78E+05 | 4.37E+05 | 5.12E+04 |
| 1796 | 0.2894 | 1.10 | 5.25E+05 | 6.01E+05 | 6.28E+05 | 5.85E+05 | 5.36E+04 | 6.35E+05 | 7.06E+05 | 5.85E+05 | 6.42E+05 | 6.07E+04 |
| 1112 | 0.2917 | 1.72 | 7.01E+05 | 5.78E+05 | 7.24E+05 | 6.68E+05 | 7.85E+04 | 1.60E+06 | 1.34E+06 | 5.11E+05 | 1.15E+06 | 5.68E+05 |
| 659 | 0.2925 | -1.25 | 1.32E+05 | 1.31E+05 | 1.73E+05 | 1.45E+05 | 2.37E+04 | 1.06E+05 | 1.59E+05 | 8.28E+04 | 1.16E+05 | 3.90E+04 |
| 2345 | 0.2929 | -1.30 | 1.32E+05 | 1.25E+05 | 7.50E+04 | 1.11E+05 | 3.11E+04 | 8.18E+04 | 7.40E+04 | 9.90E+04 | 8.49E+04 | 1.28E+04 |
| 1692 | 0.2931 | -1.21 | 1.65E+06 | 1.87E+06 | 1.19E+06 | 1.57E+06 | 3.49E+05 | 1.21E+06 | 1.32E+06 | 1.38E+06 | 1.30E+06 | 8.95E+04 |
| 2782 | 0.2935 | 1.26 | 1.20E+05 | 1.44E+05 | 1.08E+05 | 1.24E+05 | 1.87E+04 | 1.93E+05 | 1.13E+05 | 1.65E+05 | 1.57E+05 | 4.05E+04 |
| 2644 | 0.2952 | -1.15 | 6.41E+04 | 5.93E+04 | 4.62E+04 | 5.66E+04 | 9.30E+03 | 4.92E+04 | 5.35E+04 | 4.45E+04 | 4.91E+04 | 4.49E+03 |
| 2532 | 0.2954 | -1.11 | 5.24E+05 | 6.49E+05 | 5.34E+05 | 5.69E+05 | 6.93E+04 | 5.35E+05 | 5.40E+05 | 4.63E+05 | 5.13E+05 | 4.28E+04 |
| 1411 | 0.2965 | 1.24 | 6.39E+05 | 6.03E+05 | 3.98E+05 | 5.47E+05 | 1.30E+05 | 8.41E+05 | 6.21E+05 | 5.78E+05 | 6.80E+05 | 1.41E+05 |
| 2311 | 0.3007 | -1.27 | 1.72E+05 | 1.70E+05 | 1.02E+05 | 1.48E+05 | 4.01E+04 | 1.06E+05 | 1.26E+05 | 1.18E+05 | 1.17E+05 | 9.97E+03 |
| 1388 | 0.3042 | 1.22 | 4.19E+05 | 4.65E+05 | 2.56E+05 | 3.80E+05 | 1.10E+05 | 5.29E+05 | 4.35E+05 | 4.32E+05 | 4.65E+05 | 5.50E+04 |
| 524 | 0.3053 | -1.23 | 3.45E+05 | 3.73E+05 | 2.42E+05 | 3.20E+05 | 6.91E+04 | 2.82E+05 | 2.90E+05 | 2.09E+05 | 2.60E+05 | 4.45E+04 |
| 1851 | 0.3089 | 1.19 | 1.58E+05 | 1.32E+05 | 9.99E+04 | 1.30E+05 | 2.93E+04 | 1.72E+05 | 1.59E+05 | 1.31E+05 | 1.54E+05 | 2.09E+04 |
| 914 | 0.3097 | 1.50 | 6.26E+04 | 4.65E+04 | 2.94E+04 | 4.62E+04 | 1.66E+04 | 3.92E+04 | 8.14E+04 | 8.72E+04 | 6.93E+04 | 2.62E+04 |
| 5119 | 0.3159 | -1.23 | 3.83E+06 | 3.82E+06 | 4.60E+06 | 4.08E+06 | 4.50E+05 | 4.30E+06 | 2.18E+06 | 3.48E+06 | 3.32E+06 | 1.07E+06 |
| 434 | 0.3162 | 1.26 | 5.02E+04 | 6.74E+04 | 5.18E+04 | 5.65E+04 | 9.49E+03 | 5.04E+04 | 7.65E+04 | 8.73E+04 | 7.14E+04 | 1.90E+04 |
| 1117 | 0.3165 | -1.14 | 7.84E+04 | 7.85E+04 | 6.10E+04 | 7.26E+04 | 1.01E+04 | 6.61E+04 | 7.03E+04 | 5.49E+04 | 6.38E+04 | 8.00E+03 |
| 4161 | 0.3165 | -1.16 | 1.00E+06 | 1.15E+06 | 8.95E+05 | 1.01E+06 | 1.26E+05 | 1.05E+06 | 7.14E+05 | 8.63E+05 | 8.77E+05 | 1.70E+05 |
| 2119 | 0.3212 | -1.29 | 1.42E+05 | 1.43E+05 | 1.03E+05 | 1.30E+05 | 2.29E+04 | 1.29E+05 | 5.96E+04 | 1.12E+05 | 1.00E+05 | 3.63E+04 |
| 3003 | 0.3223 | 1.21 | 3.72E+05 | 3.33E+05 | 2.05E+05 | 3.03E+05 | 8.76E+04 | 3.56E+05 | 3.37E+05 | 4.04E+05 | 3.66E+05 | 3.44E+04 |
| 5121 | 0.3224 | -1.16 | 1.84E+06 | 1.68E+06 | 1.32E+06 | 1.61E+06 | 2.65E+05 | 1.50E+06 | 1.51E+06 | 1.17E+06 | 1.39E+06 | 1.95E+05 |
| 1346 | 0.3226 | 1.46 | 5.11E+04 | 6.17E+04 | 2.73E+04 | 4.67E+04 | 1.76E+04 | 1.02E+05 | 5.21E+04 | 5.05E+04 | 6.83E+04 | 2.95E+04 |
| 2748 | 0.3229 | -1.14 | 5.19E+05 | 4.77E+05 | 4.07E+05 | 4.68E+05 | 5.67E+04 | 4.56E+05 | 3.32E+05 | 4.40E+05 | 4.09E+05 | 6.74E+04 |
| 2805 | 0.3235 | -1.13 | 1.86E+06 | 1.77E+06 | 1.56E+06 | 1.73E+06 | 1.57E+05 | 1.73E+06 | 1.62E+06 | 1.22E+06 | 1.52E+06 | 2.66E+05 |
| 4226 | 0.3242 | -1.45 | 1.29E+06 | 1.59E+06 | 6.93E+05 | 1.19E+06 | 4.56E+05 | 1.07E+06 | 8.11E+05 | 5.81E+05 | 8.22E+05 | 2.46E+05 |
| 3193 | 0.3246 | 1.10 | 2.15E+06 | 2.53E+06 | 2.37E+06 | 2.35E+06 | 1.94E+05 | 2.49E+06 | 2.90E+06 | 2.33E+06 | 2.58E+06 | 2.94E+05 |
| 1065 | 0.3321 | 1.28 | 7.18E+05 | 7.42E+05 | 3.42E+05 | 6.01E+05 | 2.24E+05 | 6.52E+05 | 9.01E+05 | 7.57E+05 | 7.70E+05 | 1.25E+05 |
| 2626 | 0.3334 | 1.31 | 9.84E+04 | 1.05E+05 | 7.11E+04 | 9.14E+04 | 1.78E+04 | 1.66E+05 | 9.01E+04 | 1.02E+05 | 1.19E+05 | 4.08E+04 |
| 5124 | 0.3351 | -1.19 | 3.67E+05 | 5.62E+05 | 5.15E+05 | 4.81E+05 | 1.02E+05 | 4.62E+05 | 3.77E+05 | 3.77E+05 | 4.05E+05 | 4.91E+04 |
| 507 | 0.3424 | 1.18 | 5.35E+04 | 7.52E+04 | 8.41E+04 | 7.09E+04 | 1.57E+04 | 7.53E+04 | 7.64E+04 | 9.98E+04 | 8.38E+04 | 1.38E+04 |
| 2186 | 0.3437 | -1.11 | 3.07E+05 | 3.49E+05 | 2.57E+05 | 3.04E+05 | 4.58E+04 | 2.67E+05 | 2.75E+05 | 2.81E+05 | 2.74E+05 | 6.90E+03 |
| 1177 | 0.3443 | 1.23 | 6.22E+04 | 6.71E+04 | 3.77E+04 | 5.57E+04 | 1.57E+04 | 5.91E+04 | 8.46E+04 | 6.23E+04 | 6.87E+04 | 1.39E+04 |
| 4420 | 0.3452 | -1.29 | 7.84E+04 | 9.13E+04 | 5.54E+04 | 7.50E+04 | 1.82E+04 | 6.05E+04 | 3.75E+04 | 7.66E+04 | 5.82E+04 | 1.96E+04 |
| 1114 | 0.3463 | 1.18 | 5.41E+04 | 5.97E+04 | 3.39E+04 | 4.92E+04 | 1.36E+04 | 5.71E+04 | 6.38E+04 | 5.38E+04 | 5.82E+04 | 5.08E+03 |
| 1052 | 0.3476 | -1.26 | 5.20E+04 | 6.03E+04 | 7.24E+04 | 6.16E+04 | 1.03E+04 | 4.61E+04 | 3.06E+04 | 7.05E+04 | 4.90E+04 | 2.01E+04 |
| 4244 | 0.3485 | -1.13 | 2.92E+06 | 3.03E+06 | 2.82E+06 | 2.92E+06 | 1.02E+05 | 3.15E+06 | 2.01E+06 | 2.59E+06 | 2.58E+06 | 5.68E+05 |
| 840 | 0.3510 | -1.42 | 3.68E+04 | 1.79E+04 | 4.09E+04 | 3.18E+04 | 1.23E+04 | 1.76E+04 | 2.56E+04 | 2.42E+04 | 2.25E+04 | 4.30E+03 |
| 438 | 0.3533 | 1.30 | 3.53E+04 | 8.54E+04 | 6.51E+04 | 6.19E+04 | 2.52E+04 | 6.03E+04 | 8.76E+04 | 9.42E+04 | 8.07E+04 | 1.80E+04 |
| 408 | 0.3542 | 1.27 | 9.86E+05 | 9.62E+05 | 9.15E+05 | 9.55E+05 | 3.60E+04 | 1.56E+06 | 1.28E+06 | 8.07E+05 | 1.22E+06 | 3.81E+05 |
| 396 | 0.3589 | -1.27 | 8.61E+04 | 1.22E+05 | 1.23E+05 | 1.10E+05 | 2.09E+04 | 5.21E+04 | 1.24E+05 | 8.48E+04 | 8.68E+04 | 3.58E+04 |
| 1051 | 0.3601 | 1.24 | 4.06E+05 | 6.36E+05 | 4.10E+05 | 4.84E+05 | 1.32E+05 | 6.84E+05 | 4.37E+05 | 6.78E+05 | 6.00E+05 | 1.41E+05 |
| 2328 | 0.3608 | -1.22 | 1.20E+05 | 8.97E+04 | 6.80E+04 | 9.26E+04 | 2.62E+04 | 6.92E+04 | 8.21E+04 | 7.59E+04 | 7.57E+04 | 6.42E+03 |
| 4271 | 0.3639 | -1.17 | 1.10E+06 | 1.12E+06 | 8.45E+05 | 1.02E+06 | 1.54E+05 | 1.04E+06 | 6.52E+05 | 9.15E+05 | 8.71E+05 | 2.00E+05 |
| 2548 | 0.3656 | -1.56 | 2.91E+05 | 3.41E+05 | 1.17E+05 | 2.50E+05 | 1.18E+05 | 1.98E+05 | 1.43E+05 | 1.41E+05 | 1.61E+05 | 3.25E+04 |
| 5088 | 0.3669 | 1.18 | 3.81E+06 | 2.82E+06 | 2.07E+06 | 2.90E+06 | 8.71E+05 | 3.64E+06 | 2.96E+06 | 3.66E+06 | 3.42E+06 | 4.00E+05 |
| 1328 | 0.3685 | -1.08 | 7.70E+05 | 9.06E+05 | 8.39E+05 | 8.38E+05 | 6.77E+04 | 8.19E+05 | 8.26E+05 | 6.83E+05 | 7.76E+05 | 8.08E+04 |
| 5136 | 0.3710 | -1.49 | 2.51E+05 | 5.11E+05 | 1.99E+05 | 3.21E+05 | 1.67E+05 | 2.86E+05 | 2.20E+05 | 1.41E+05 | 2.16E+05 | 7.23E+04 |
| 2137 | 0.3712 | 1.39 | 2.72E+04 | 2.22E+04 | 2.50E+04 | 2.48E+04 | 2.54E+03 | 2.91E+04 | 2.19E+04 | 5.24E+04 | 3.45E+04 | 1.60E+04 |
| 3935 | 0.3731 | -1.22 | 2.00E+05 | 1.85E+05 | 2.49E+05 | 2.11E+05 | 3.34E+04 | 1.77E+05 | 2.31E+05 | 1.12E+05 | 1.74E+05 | 5.98E+04 |
| 5100 | 0.3748 | -1.15 | 3.37E+04 | 2.47E+04 | 3.40E+04 | 3.08E+04 | 5.28E+03 | 2.26E+04 | 3.07E+04 | 2.70E+04 | 2.68E+04 | 4.05E+03 |
| 743 | 0.3785 | 1.11 | 2.58E+05 | 3.13E+05 | 2.65E+05 | 2.78E+05 | 2.98E+04 | 3.52E+05 | 2.69E+05 | 3.03E+05 | 3.08E+05 | 4.19E+04 |
| 1518 | 0.3789 | -1.13 | 1.31E+05 | 1.29E+05 | 1.40E+05 | 1.33E+05 | 6.09E+03 | 1.06E+05 | 1.52E+05 | 9.76E+04 | 1.18E+05 | 2.92E+04 |
| 5132 | 0.3791 | 1.09 | 1.79E+06 | 1.84E+06 | 1.44E+06 | 1.69E+06 | 2.19E+05 | 2.03E+06 | 1.77E+06 | 1.73E+06 | 1.84E+06 | 1.62E+05 |
| 4169 | 0.3847 | -1.37 | 6.19E+05 | 9.03E+05 | 5.14E+05 | 6.79E+05 | 2.01E+05 | 7.67E+05 | 5.31E+05 | 1.87E+05 | 4.95E+05 | 2.91E+05 |
| 1465 | 0.3897 | 1.08 | 2.34E+05 | 2.54E+05 | 1.96E+05 | 2.28E+05 | 2.96E+04 | 2.66E+05 | 2.47E+05 | 2.29E+05 | 2.47E+05 | 1.82E+04 |
| 2573 | 0.3903 | -1.26 | 1.02E+05 | 1.45E+05 | 1.29E+05 | 1.25E+05 | 2.18E+04 | 5.32E+04 | 8.60E+04 | 1.59E+05 | 9.93E+04 | 5.40E+04 |
| 2093 | 0.3904 | -1.12 | 4.50E+05 | 3.93E+05 | 4.83E+05 | 4.42E+05 | 4.55E+04 | 3.44E+05 | 4.86E+05 | 3.56E+05 | 3.96E+05 | 7.86E+04 |
| 3957 | 0.3907 | 1.31 | 1.92E+05 | 2.56E+05 | 2.25E+05 | 2.24E+05 | 3.17E+04 | 3.11E+05 | 1.83E+05 | 3.86E+05 | 2.93E+05 | 1.03E+05 |
| 2334 | 0.3933 | 1.22 | 1.01E+05 | 1.56E+05 | 7.46E+04 | 1.10E+05 | 4.13E+04 | 1.11E+05 | 1.62E+05 | 1.29E+05 | 1.34E+05 | 2.61E+04 |
| 1885 | 0.3934 | -1.20 | 1.97E+06 | 2.00E+06 | 1.22E+06 | 1.73E+06 | 4.39E+05 | 1.52E+06 | 1.48E+06 | 1.33E+06 | 1.44E+06 | 1.02E+05 |
| 566 | 0.3971 | -1.43 | 2.34E+05 | 2.01E+05 | 5.07E+05 | 3.14E+05 | 1.68E+05 | 2.01E+05 | 2.31E+05 | 2.25E+05 | 2.19E+05 | 1.56E+04 |
| 2787 | 0.4037 | 1.17 | 3.93E+05 | 2.46E+05 | 2.00E+05 | 2.80E+05 | 1.01E+05 | 3.59E+05 | 3.22E+05 | 2.99E+05 | 3.26E+05 | 3.03E+04 |
| 474 | 0.4062 | -1.18 | 1.26E+05 | 1.28E+05 | 1.94E+05 | 1.49E+05 | 3.84E+04 | 1.23E+05 | 1.45E+05 | 1.13E+05 | 1.27E+05 | 1.63E+04 |
| 1340 | 0.4063 | 1.24 | 7.00E+04 | 6.32E+04 | 2.38E+04 | 5.23E+04 | 2.49E+04 | 6.91E+04 | 6.36E+04 | 6.26E+04 | 6.51E+04 | 3.53E+03 |
| 2726 | 0.4077 | 1.13 | 3.29E+05 | 3.32E+05 | 2.10E+05 | 2.90E+05 | 6.95E+04 | 3.15E+05 | 3.54E+05 | 3.16E+05 | 3.28E+05 | 2.20E+04 |
| 3288 | 0.4163 | 1.08 | 4.98E+06 | 4.65E+06 | 4.23E+06 | 4.62E+06 | 3.78E+05 | 5.51E+06 | 5.18E+06 | 4.34E+06 | 5.01E+06 | 6.04E+05 |
| 982 | 0.4173 | -1.29 | 8.87E+04 | 6.97E+04 | 1.46E+05 | 1.02E+05 | 3.98E+04 | 7.31E+04 | 9.59E+04 | 6.67E+04 | 7.85E+04 | 1.54E+04 |
| 580 | 0.4193 | -1.17 | 4.14E+04 | 4.21E+04 | 3.09E+04 | 3.81E+04 | 6.29E+03 | 2.75E+04 | 4.39E+04 | 2.66E+04 | 3.27E+04 | 9.77E+03 |
| 324 | 0.4237 | 1.19 | 1.82E+04 | 1.73E+04 | 1.05E+05 | 4.69E+04 | 5.05E+04 | 4.87E+04 | 6.96E+04 | 4.92E+04 | 5.58E+04 | 1.19E+04 |
| 5069 | 0.4319 | -1.26 | 7.45E+04 | 8.92E+04 | 1.43E+05 | 1.02E+05 | 3.61E+04 | 1.05E+05 | 7.77E+04 | 6.06E+04 | 8.10E+04 | 2.22E+04 |
| 958 | 0.4346 | 1.14 | 4.04E+05 | 3.38E+05 | 3.05E+05 | 3.49E+05 | 5.02E+04 | 4.55E+05 | 4.20E+05 | 3.13E+05 | 3.96E+05 | 7.40E+04 |
| 929 | 0.4346 | 1.24 | 5.77E+04 | 6.32E+04 | 3.78E+04 | 5.29E+04 | 1.34E+04 | 4.59E+04 | 8.39E+04 | 6.63E+04 | 6.54E+04 | 1.90E+04 |
| 1877 | 0.4347 | -1.16 | 1.07E+05 | 8.69E+04 | 1.02E+05 | 9.87E+04 | 1.05E+04 | 8.49E+04 | 1.14E+05 | 5.58E+04 | 8.49E+04 | 2.91E+04 |
| 3806 | 0.4352 | -1.23 | 8.56E+05 | 5.47E+05 | 4.94E+05 | 6.32E+05 | 1.95E+05 | 4.76E+05 | 3.87E+05 | 6.81E+05 | 5.15E+05 | 1.51E+05 |
| 1537 | 0.4370 | -1.06 | 4.42E+05 | 4.11E+05 | 4.62E+05 | 4.39E+05 | 2.55E+04 | 4.59E+05 | 4.06E+05 | 3.81E+05 | 4.16E+05 | 4.01E+04 |
| 1807 | 0.4401 | 1.16 | 1.50E+06 | 1.68E+06 | 1.48E+06 | 1.55E+06 | 1.08E+05 | 2.15E+06 | 1.34E+06 | 1.89E+06 | 1.79E+06 | 4.18E+05 |
| 4201 | 0.4415 | -1.21 | 1.25E+05 | 1.44E+05 | 1.16E+05 | 1.28E+05 | 1.45E+04 | 1.22E+05 | 1.44E+05 | 5.29E+04 | 1.06E+05 | 4.74E+04 |
| 2751 | 0.4419 | -1.11 | 2.43E+05 | 2.09E+05 | 1.93E+05 | 2.15E+05 | 2.54E+04 | 2.35E+05 | 1.87E+05 | 1.60E+05 | 1.94E+05 | 3.83E+04 |
| 2301 | 0.4421 | -1.15 | 1.35E+05 | 1.22E+05 | 1.01E+05 | 1.19E+05 | 1.73E+04 | 1.08E+05 | 7.39E+04 | 1.29E+05 | 1.04E+05 | 2.80E+04 |
| 5148 | 0.4497 | 1.21 | 5.54E+05 | 4.06E+05 | 1.77E+05 | 3.79E+05 | 1.90E+05 | 4.04E+05 | 5.16E+05 | 4.56E+05 | 4.59E+05 | 5.58E+04 |
| 478 | 0.4530 | -1.27 | 1.71E+05 | 1.28E+05 | 2.77E+05 | 1.92E+05 | 7.66E+04 | 1.34E+05 | 1.54E+05 | 1.64E+05 | 1.51E+05 | 1.54E+04 |
| 2456 | 0.4543 | 1.20 | 6.65E+04 | 9.53E+04 | 4.93E+04 | 7.04E+04 | 2.33E+04 | 1.09E+05 | 6.55E+04 | 7.99E+04 | 8.47E+04 | 2.21E+04 |
| 1516 | 0.4544 | -1.13 | 1.64E+05 | 1.58E+05 | 1.37E+05 | 1.53E+05 | 1.44E+04 | 1.41E+05 | 1.70E+05 | 9.51E+04 | 1.35E+05 | 3.76E+04 |
| 2736 | 0.4552 | 1.16 | 4.38E+05 | 5.03E+05 | 2.50E+05 | 3.97E+05 | 1.32E+05 | 5.14E+05 | 4.53E+05 | 4.11E+05 | 4.59E+05 | 5.20E+04 |
| 2899 | 0.4566 | 1.20 | 5.96E+04 | 8.31E+04 | 1.24E+05 | 8.89E+04 | 3.26E+04 | 1.18E+05 | 7.91E+04 | 1.23E+05 | 1.07E+05 | 2.42E+04 |
| 653 | 0.4575 | -1.27 | 9.55E+04 | 7.22E+04 | 1.50E+05 | 1.06E+05 | 3.97E+04 | 6.09E+04 | 7.74E+04 | 1.11E+05 | 8.31E+04 | 2.56E+04 |
| 690 | 0.4577 | 1.12 | 1.20E+05 | 1.34E+05 | 8.13E+04 | 1.12E+05 | 2.73E+04 | 1.16E+05 | 1.35E+05 | 1.23E+05 | 1.25E+05 | 9.51E+03 |
| 2066 | 0.4580 | -1.16 | 2.61E+05 | 2.65E+05 | 1.68E+05 | 2.31E+05 | 5.46E+04 | 1.88E+05 | 2.16E+05 | 1.95E+05 | 2.00E+05 | 1.46E+04 |
| 1671 | 0.4633 | -1.09 | 1.13E+06 | 9.86E+05 | 8.40E+05 | 9.85E+05 | 1.45E+05 | 8.43E+05 | 9.84E+05 | 8.91E+05 | 9.06E+05 | 7.17E+04 |
| 3960 | 0.4638 | 1.18 | 2.19E+05 | 2.10E+05 | 1.20E+05 | 1.83E+05 | 5.46E+04 | 2.61E+05 | 2.14E+05 | 1.71E+05 | 2.16E+05 | 4.51E+04 |
| 2426 | 0.4687 | 1.15 | 4.86E+04 | 6.45E+04 | 3.87E+04 | 5.06E+04 | 1.30E+04 | 6.34E+04 | 4.59E+04 | 6.51E+04 | 5.81E+04 | 1.06E+04 |
| 1435 | 0.4702 | -1.19 | 1.73E+05 | 1.26E+05 | 1.02E+05 | 1.34E+05 | 3.61E+04 | 1.09E+05 | 1.44E+05 | 8.32E+04 | 1.12E+05 | 3.07E+04 |
| 829 | 0.4779 | -1.08 | 4.67E+05 | 4.38E+05 | 5.43E+05 | 4.83E+05 | 5.40E+04 | 3.78E+05 | 5.08E+05 | 4.50E+05 | 4.46E+05 | 6.54E+04 |
| 1679 | 0.4788 | -1.16 | 4.02E+05 | 3.22E+05 | 4.94E+05 | 4.06E+05 | 8.60E+04 | 2.42E+05 | 3.82E+05 | 4.22E+05 | 3.49E+05 | 9.45E+04 |
| 2721 | 0.4796 | 1.10 | 1.65E+05 | 2.28E+05 | 1.33E+05 | 1.75E+05 | 4.84E+04 | 1.92E+05 | 2.01E+05 | 1.87E+05 | 1.93E+05 | 6.99E+03 |
| 2973 | 0.4801 | 1.41 | 1.01E+05 | 8.92E+04 | 1.09E+05 | 9.98E+04 | 1.01E+04 | 6.90E+04 | 1.50E+05 | 2.03E+05 | 1.41E+05 | 6.75E+04 |
| 1789 | 0.4816 | 1.13 | 8.66E+05 | 1.18E+06 | 6.84E+05 | 9.10E+05 | 2.50E+05 | 1.17E+06 | 8.84E+05 | 1.02E+06 | 1.02E+06 | 1.42E+05 |
| 1864 | 0.4822 | -1.20 | 1.14E+05 | 1.38E+05 | 2.16E+05 | 1.56E+05 | 5.37E+04 | 1.32E+05 | 1.24E+05 | 1.33E+05 | 1.30E+05 | 4.86E+03 |
| 477 | 0.4831 | -1.21 | 1.52E+05 | 1.27E+05 | 2.30E+05 | 1.70E+05 | 5.35E+04 | 1.18E+05 | 1.11E+05 | 1.92E+05 | 1.41E+05 | 4.49E+04 |
| 3946 | 0.4853 | 1.08 | 1.16E+06 | 1.12E+06 | 8.44E+05 | 1.04E+06 | 1.70E+05 | 1.23E+06 | 1.06E+06 | 1.08E+06 | 1.12E+06 | 9.06E+04 |
| 1720 | 0.4868 | -1.19 | 2.67E+06 | 3.10E+06 | 1.75E+06 | 2.51E+06 | 6.88E+05 | 2.55E+06 | 2.16E+06 | 1.59E+06 | 2.10E+06 | 4.82E+05 |
| 2275 | 0.4886 | 1.09 | 3.70E+06 | 3.92E+06 | 2.72E+06 | 3.45E+06 | 6.37E+05 | 3.99E+06 | 3.92E+06 | 3.36E+06 | 3.76E+06 | 3.44E+05 |
| 721 | 0.4887 | -1.16 | 5.24E+04 | 3.49E+04 | 5.31E+04 | 4.68E+04 | 1.03E+04 | 3.54E+04 | 5.26E+04 | 3.30E+04 | 4.03E+04 | 1.07E+04 |
| 2812 | 0.4987 | -1.06 | 1.52E+05 | 1.50E+05 | 1.27E+05 | 1.43E+05 | 1.39E+04 | 1.49E+05 | 1.31E+05 | 1.24E+05 | 1.35E+05 | 1.31E+04 |
| 567 | 0.4991 | -1.23 | 2.03E+05 | 1.93E+05 | 3.71E+05 | 2.55E+05 | 9.98E+04 | 1.85E+05 | 2.33E+05 | 2.07E+05 | 2.08E+05 | 2.39E+04 |
| 2089 | 0.5005 | -1.11 | 6.84E+05 | 6.93E+05 | 6.45E+05 | 6.74E+05 | 2.54E+04 | 6.65E+05 | 7.41E+05 | 4.22E+05 | 6.09E+05 | 1.67E+05 |
| 5098 | 0.5047 | 1.09 | 8.69E+04 | 6.10E+04 | 5.22E+04 | 6.67E+04 | 1.80E+04 | 7.27E+04 | 7.66E+04 | 6.96E+04 | 7.30E+04 | 3.52E+03 |
| 5145 | 0.5100 | -1.20 | 1.31E+06 | 1.31E+06 | 2.17E+06 | 1.60E+06 | 4.98E+05 | 1.41E+06 | 1.71E+06 | 8.73E+05 | 1.33E+06 | 4.25E+05 |
| 2877 | 0.5140 | 1.13 | 8.02E+05 | 7.94E+05 | 4.16E+05 | 6.71E+05 | 2.20E+05 | 6.73E+05 | 7.47E+05 | 8.60E+05 | 7.60E+05 | 9.41E+04 |
| 1891 | 0.5186 | -1.16 | 1.02E+05 | 7.75E+04 | 1.09E+05 | 9.61E+04 | 1.65E+04 | 7.49E+04 | 1.27E+05 | 4.77E+04 | 8.32E+04 | 4.04E+04 |
| 1926 | 0.5222 | 1.11 | 6.65E+04 | 3.96E+04 | 6.87E+04 | 5.83E+04 | 1.62E+04 | 5.86E+04 | 7.14E+04 | 6.41E+04 | 6.47E+04 | 6.43E+03 |
| 2911 | 0.5281 | -1.10 | 6.24E+04 | 7.74E+04 | 6.14E+04 | 6.71E+04 | 8.97E+03 | 7.65E+04 | 4.84E+04 | 5.84E+04 | 6.11E+04 | 1.42E+04 |
| 556 | 0.5282 | -1.43 | 6.63E+04 | 5.23E+04 | 1.61E+05 | 9.31E+04 | 5.90E+04 | 6.04E+04 | 7.36E+04 | 6.11E+04 | 6.50E+04 | 7.43E+03 |
| 779 | 0.5286 | 1.19 | 2.93E+04 | 2.58E+04 | 2.82E+04 | 2.78E+04 | 1.78E+03 | 2.22E+04 | 3.35E+04 | 4.32E+04 | 3.30E+04 | 1.05E+04 |
| 1492 | 0.5445 | -1.16 | 6.34E+04 | 7.16E+04 | 4.32E+04 | 5.94E+04 | 1.46E+04 | 4.14E+04 | 3.91E+04 | 7.34E+04 | 5.13E+04 | 1.92E+04 |
| 1583 | 0.5458 | 1.14 | 1.03E+05 | 1.15E+05 | 7.44E+04 | 9.72E+04 | 2.06E+04 | 1.24E+05 | 8.18E+04 | 1.26E+05 | 1.11E+05 | 2.50E+04 |
| 2538 | 0.5499 | -1.05 | 1.98E+05 | 1.96E+05 | 1.73E+05 | 1.89E+05 | 1.39E+04 | 2.08E+05 | 1.66E+05 | 1.65E+05 | 1.80E+05 | 2.44E+04 |
| 2078 | 0.5522 | 1.11 | 1.51E+05 | 1.81E+05 | 1.61E+05 | 1.64E+05 | 1.53E+04 | 1.47E+05 | 1.71E+05 | 2.32E+05 | 1.83E+05 | 4.37E+04 |
| 1203 | 0.5546 | -1.14 | 1.28E+06 | 1.62E+06 | 1.01E+06 | 1.30E+06 | 3.06E+05 | 1.30E+06 | 1.29E+06 | 8.44E+05 | 1.15E+06 | 2.62E+05 |
| 1226 | 0.5608 | -1.15 | 4.10E+05 | 4.52E+05 | 2.87E+05 | 3.83E+05 | 8.57E+04 | 4.00E+05 | 3.90E+05 | 2.12E+05 | 3.34E+05 | 1.06E+05 |
| 974 | 0.5618 | -1.19 | 2.29E+04 | 2.50E+04 | 4.23E+04 | 3.01E+04 | 1.06E+04 | 2.13E+04 | 1.59E+04 | 3.87E+04 | 2.53E+04 | 1.19E+04 |
| 639 | 0.5620 | 1.09 | 5.47E+05 | 5.00E+05 | 3.42E+05 | 4.63E+05 | 1.08E+05 | 4.99E+05 | 5.64E+05 | 4.49E+05 | 5.04E+05 | 5.76E+04 |
| 614 | 0.5621 | 1.09 | 8.15E+04 | 8.39E+04 | 7.88E+04 | 8.14E+04 | 2.57E+03 | 1.08E+05 | 8.52E+04 | 7.29E+04 | 8.87E+04 | 1.78E+04 |
| 1289 | 0.5641 | -1.09 | 7.44E+04 | 7.05E+04 | 5.23E+04 | 6.57E+04 | 1.18E+04 | 5.44E+04 | 5.66E+04 | 6.93E+04 | 6.01E+04 | 8.07E+03 |
| 1763 | 0.5738 | 1.11 | 1.54E+05 | 1.94E+05 | 1.03E+05 | 1.50E+05 | 4.56E+04 | 1.91E+05 | 1.36E+05 | 1.74E+05 | 1.67E+05 | 2.82E+04 |
| 4770 | 0.5739 | -1.11 | 2.33E+05 | 2.19E+05 | 2.30E+05 | 2.27E+05 | 7.54E+03 | 1.00E+05 | 2.36E+05 | 2.77E+05 | 2.04E+05 | 9.27E+04 |
| 1779 | 0.5741 | 1.10 | 5.86E+05 | 5.12E+05 | 3.79E+05 | 4.92E+05 | 1.05E+05 | 6.06E+05 | 5.81E+05 | 4.37E+05 | 5.41E+05 | 9.15E+04 |
| 1208 | 0.5760 | -1.10 | 4.51E+05 | 5.41E+05 | 3.79E+05 | 4.57E+05 | 8.15E+04 | 4.67E+05 | 4.73E+05 | 3.01E+05 | 4.14E+05 | 9.75E+04 |
| 3778 | 0.5770 | -1.08 | 8.94E+05 | 8.48E+05 | 8.81E+05 | 8.74E+05 | 2.40E+04 | 8.87E+05 | 9.90E+05 | 5.43E+05 | 8.07E+05 | 2.34E+05 |
| 1441 | 0.5802 | -1.07 | 7.96E+04 | 1.02E+05 | 9.50E+04 | 9.23E+04 | 1.16E+04 | 8.87E+04 | 7.10E+04 | 9.86E+04 | 8.61E+04 | 1.40E+04 |
| 2661 | 0.5836 | 1.14 | 7.44E+05 | 7.13E+05 | 3.47E+05 | 6.01E+05 | 2.21E+05 | 5.53E+05 | 8.67E+05 | 6.34E+05 | 6.85E+05 | 1.63E+05 |
| 733 | 0.5850 | -1.07 | 2.27E+05 | 2.24E+05 | 1.83E+05 | 2.11E+05 | 2.48E+04 | 2.28E+05 | 2.02E+05 | 1.62E+05 | 1.97E+05 | 3.30E+04 |
| 2845 | 0.5865 | 1.32 | 5.09E+04 | 5.06E+04 | 6.21E+04 | 5.45E+04 | 6.53E+03 | 1.09E+05 | 3.73E+04 | 6.91E+04 | 7.18E+04 | 3.59E+04 |
| 2072 | 0.5868 | 1.12 | 9.75E+04 | 1.09E+05 | 5.55E+04 | 8.73E+04 | 2.81E+04 | 1.20E+05 | 8.11E+04 | 9.23E+04 | 9.78E+04 | 2.00E+04 |
| 4102 | 0.5911 | 1.19 | 2.48E+05 | 4.36E+05 | 2.06E+05 | 2.97E+05 | 1.23E+05 | 2.80E+05 | 2.65E+05 | 5.18E+05 | 3.54E+05 | 1.42E+05 |
| 435 | 0.5941 | 1.14 | 6.52E+04 | 1.85E+05 | 1.36E+05 | 1.29E+05 | 6.01E+04 | 1.19E+05 | 1.30E+05 | 1.92E+05 | 1.47E+05 | 3.92E+04 |
| 2580 | 0.5973 | -1.12 | 8.58E+04 | 1.03E+05 | 1.29E+05 | 1.06E+05 | 2.20E+04 | 9.21E+04 | 5.58E+04 | 1.36E+05 | 9.46E+04 | 4.01E+04 |
| 1766 | 0.6026 | 1.11 | 5.45E+05 | 5.47E+05 | 4.15E+05 | 5.02E+05 | 7.55E+04 | 7.26E+05 | 4.97E+05 | 4.57E+05 | 5.60E+05 | 1.45E+05 |
| 987 | 0.6042 | 1.21 | 1.09E+05 | 1.03E+05 | 8.67E+04 | 9.95E+04 | 1.17E+04 | 7.28E+04 | 1.23E+05 | 1.65E+05 | 1.20E+05 | 4.61E+04 |
| 1238 | 0.6048 | -1.06 | 7.72E+05 | 6.93E+05 | 9.16E+05 | 7.94E+05 | 1.13E+05 | 6.92E+05 | 7.41E+05 | 8.16E+05 | 7.50E+05 | 6.24E+04 |
| 1259 | 0.6051 | -1.20 | 1.22E+06 | 1.55E+06 | 7.18E+05 | 1.16E+06 | 4.20E+05 | 1.35E+06 | 1.01E+06 | 5.56E+05 | 9.73E+05 | 4.00E+05 |
| 3798 | 0.6055 | -1.16 | 1.37E+05 | 2.15E+05 | 1.16E+05 | 1.56E+05 | 5.24E+04 | 1.15E+05 | 1.90E+05 | 9.87E+04 | 1.35E+05 | 4.88E+04 |
| 1631 | 0.6101 | 1.37 | 1.34E+05 | 1.29E+05 | 1.11E+05 | 1.25E+05 | 1.20E+04 | 1.27E+05 | 9.23E+04 | 2.93E+05 | 1.71E+05 | 1.07E+05 |
| 1636 | 0.6109 | 1.19 | 2.19E+05 | 2.77E+05 | 2.05E+05 | 2.34E+05 | 3.81E+04 | 2.76E+05 | 1.75E+05 | 3.85E+05 | 2.79E+05 | 1.05E+05 |
| 1290 | 0.6116 | -1.13 | 1.18E+05 | 1.72E+05 | 9.82E+04 | 1.29E+05 | 3.80E+04 | 1.20E+05 | 1.06E+05 | 1.19E+05 | 1.15E+05 | 7.71E+03 |
| 2966 | 0.6143 | -1.09 | 2.38E+05 | 2.16E+05 | 2.76E+05 | 2.43E+05 | 3.05E+04 | 2.18E+05 | 3.20E+05 | 1.30E+05 | 2.23E+05 | 9.49E+04 |
| 2794 | 0.6160 | 1.07 | 7.06E+04 | 9.38E+04 | 5.63E+04 | 7.35E+04 | 1.89E+04 | 8.55E+04 | 7.62E+04 | 7.36E+04 | 7.84E+04 | 6.24E+03 |
| 5130 | 0.6179 | 1.06 | 1.79E+06 | 1.72E+06 | 1.23E+06 | 1.58E+06 | 3.04E+05 | 1.68E+06 | 1.81E+06 | 1.53E+06 | 1.67E+06 | 1.44E+05 |
| 406 | 0.6200 | 1.45 | 7.61E+04 | 9.12E+04 | 1.76E+05 | 1.15E+05 | 5.39E+04 | 1.16E+05 | 7.53E+04 | 3.08E+05 | 1.66E+05 | 1.25E+05 |
| 737 | 0.6264 | 1.06 | 1.03E+05 | 1.34E+05 | 7.70E+04 | 1.05E+05 | 2.83E+04 | 1.13E+05 | 1.12E+05 | 1.08E+05 | 1.11E+05 | 3.03E+03 |
| 622 | 0.6265 | 1.09 | 7.96E+04 | 6.89E+04 | 5.35E+04 | 6.74E+04 | 1.31E+04 | 6.93E+04 | 6.08E+04 | 9.04E+04 | 7.35E+04 | 1.53E+04 |
| 2243 | 0.6274 | -1.28 | 1.32E+05 | 8.85E+04 | 4.36E+04 | 8.80E+04 | 4.43E+04 | 3.86E+04 | 6.39E+04 | 1.04E+05 | 6.90E+04 | 3.32E+04 |
| 729 | 0.6279 | -1.06 | 8.22E+04 | 7.43E+04 | 7.64E+04 | 7.76E+04 | 4.11E+03 | 7.09E+04 | 9.16E+04 | 5.82E+04 | 7.35E+04 | 1.69E+04 |
| 2455 | 0.6280 | 1.12 | 1.07E+05 | 1.24E+05 | 6.79E+04 | 9.97E+04 | 2.90E+04 | 1.48E+05 | 9.78E+04 | 9.06E+04 | 1.12E+05 | 3.11E+04 |
| 1329 | 0.6288 | 1.06 | 8.18E+05 | 8.57E+05 | 6.45E+05 | 7.73E+05 | 1.13E+05 | 9.02E+05 | 8.43E+05 | 7.10E+05 | 8.18E+05 | 9.83E+04 |
| 1274 | 0.6308 | 1.07 | 6.03E+05 | 6.40E+05 | 3.72E+05 | 5.38E+05 | 1.45E+05 | 5.12E+05 | 6.05E+05 | 6.15E+05 | 5.77E+05 | 5.67E+04 |
| 2397 | 0.6356 | 1.06 | 3.58E+05 | 3.80E+05 | 2.36E+05 | 3.25E+05 | 7.78E+04 | 3.33E+05 | 3.35E+05 | 3.63E+05 | 3.44E+05 | 1.65E+04 |
| 486 | 0.6369 | -1.12 | 7.92E+04 | 8.21E+04 | 1.35E+05 | 9.89E+04 | 3.16E+04 | 6.43E+04 | 6.06E+04 | 1.39E+05 | 8.79E+04 | 4.42E+04 |
| 382 | 0.6377 | -1.16 | 5.12E+04 | 4.18E+04 | 8.36E+04 | 5.89E+04 | 2.19E+04 | 4.92E+04 | 5.55E+04 | 4.75E+04 | 5.07E+04 | 4.22E+03 |
| 669 | 0.6387 | 1.13 | 7.24E+04 | 7.69E+04 | 4.95E+04 | 6.63E+04 | 1.47E+04 | 8.78E+04 | 8.46E+04 | 5.18E+04 | 7.47E+04 | 1.99E+04 |
| 563 | 0.6396 | -1.37 | 1.58E+05 | 1.06E+05 | 3.67E+05 | 2.11E+05 | 1.38E+05 | 1.24E+05 | 1.87E+05 | 1.49E+05 | 1.53E+05 | 3.19E+04 |
| 1056 | 0.6404 | 1.07 | 3.07E+04 | 2.85E+04 | 1.96E+04 | 2.63E+04 | 5.83E+03 | 2.73E+04 | 2.50E+04 | 3.16E+04 | 2.80E+04 | 3.33E+03 |
| 976 | 0.6419 | -1.09 | 7.38E+04 | 5.27E+04 | 6.47E+04 | 6.37E+04 | 1.06E+04 | 5.38E+04 | 3.81E+04 | 8.43E+04 | 5.87E+04 | 2.35E+04 |
| 732 | 0.6442 | -1.21 | 3.72E+05 | 2.11E+05 | 5.08E+05 | 3.63E+05 | 1.49E+05 | 3.02E+05 | 3.49E+05 | 2.50E+05 | 3.00E+05 | 4.95E+04 |
| 3791 | 0.6443 | 1.13 | 1.80E+05 | 2.08E+05 | 1.14E+05 | 1.67E+05 | 4.81E+04 | 2.44E+05 | 1.36E+05 | 1.87E+05 | 1.89E+05 | 5.43E+04 |
| 5085 | 0.6455 | 1.08 | 2.39E+06 | 1.41E+06 | 1.23E+06 | 1.68E+06 | 6.22E+05 | 2.11E+06 | 1.72E+06 | 1.59E+06 | 1.80E+06 | 2.72E+05 |
| 5104 | 0.6466 | -1.03 | 5.87E+05 | 6.08E+05 | 6.28E+05 | 6.08E+05 | 2.03E+04 | 6.50E+05 | 6.10E+05 | 5.04E+05 | 5.88E+05 | 7.52E+04 |
| 3247 | 0.6469 | -1.36 | 6.00E+05 | 7.88E+05 | 2.29E+05 | 5.39E+05 | 2.84E+05 | 4.05E+05 | 4.53E+05 | 3.33E+05 | 3.97E+05 | 6.06E+04 |
| 376 | 0.6482 | 1.06 | 7.66E+04 | 6.53E+04 | 4.83E+04 | 6.34E+04 | 1.42E+04 | 6.15E+04 | 7.45E+04 | 6.55E+04 | 6.71E+04 | 6.66E+03 |
| 4253 | 0.6505 | -1.05 | 1.01E+06 | 9.59E+05 | 9.27E+05 | 9.66E+05 | 4.26E+04 | 1.19E+06 | 7.17E+05 | 8.42E+05 | 9.17E+05 | 2.46E+05 |
| 1449 | 0.6507 | 1.19 | 6.33E+04 | 4.46E+04 | 7.29E+04 | 6.02E+04 | 1.44E+04 | 5.07E+04 | 5.65E+04 | 1.08E+05 | 7.16E+04 | 3.14E+04 |
| 3772 | 0.6514 | -1.25 | 1.00E+05 | 8.61E+04 | 3.86E+04 | 7.50E+04 | 3.23E+04 | 6.14E+04 | 6.01E+04 | 5.84E+04 | 6.00E+04 | 1.50E+03 |
| 2107 | 0.6525 | 1.07 | 3.11E+05 | 3.43E+05 | 3.43E+05 | 3.32E+05 | 1.86E+04 | 3.66E+05 | 4.19E+05 | 2.84E+05 | 3.56E+05 | 6.80E+04 |
| 833 | 0.6544 | 1.06 | 1.10E+05 | 1.10E+05 | 7.47E+04 | 9.81E+04 | 2.02E+04 | 1.15E+05 | 9.65E+04 | 9.93E+04 | 1.04E+05 | 9.90E+03 |
| 668 | 0.6633 | -1.08 | 1.28E+05 | 1.12E+05 | 8.52E+04 | 1.08E+05 | 2.15E+04 | 1.30E+05 | 8.87E+04 | 8.07E+04 | 9.99E+04 | 2.67E+04 |
| 2914 | 0.6683 | 1.06 | 1.51E+05 | 1.58E+05 | 5.12E+04 | 1.20E+05 | 5.96E+04 | 1.23E+05 | 1.35E+05 | 1.23E+05 | 1.27E+05 | 6.79E+03 |
| 1055 | 0.6695 | 1.11 | 8.87E+04 | 1.09E+05 | 7.12E+04 | 8.97E+04 | 1.89E+04 | 9.03E+04 | 7.61E+04 | 1.33E+05 | 9.99E+04 | 2.98E+04 |
| 939 | 0.6727 | -1.06 | 3.52E+05 | 3.14E+05 | 2.52E+05 | 3.06E+05 | 5.04E+04 | 3.20E+05 | 2.94E+05 | 2.51E+05 | 2.88E+05 | 3.46E+04 |
| 1172 | 0.6733 | 1.08 | 5.73E+04 | 6.62E+04 | 3.15E+04 | 5.17E+04 | 1.80E+04 | 5.26E+04 | 6.83E+04 | 4.69E+04 | 5.59E+04 | 1.10E+04 |
| 918 | 0.6736 | 1.17 | 6.94E+04 | 6.74E+04 | 6.73E+04 | 6.80E+04 | 1.19E+03 | 6.53E+04 | 5.55E+04 | 1.19E+05 | 7.98E+04 | 3.39E+04 |
| 534 | 0.6769 | 1.08 | 3.04E+04 | 3.31E+04 | 2.49E+04 | 2.95E+04 | 4.18E+03 | 2.60E+04 | 3.93E+04 | 2.99E+04 | 3.17E+04 | 6.84E+03 |
| 1841 | 0.6800 | 1.07 | 1.12E+05 | 1.55E+05 | 1.12E+05 | 1.26E+05 | 2.47E+04 | 1.45E+05 | 1.07E+05 | 1.55E+05 | 1.35E+05 | 2.55E+04 |
| 458 | 0.6806 | -1.05 | 4.54E+04 | 4.90E+04 | 4.92E+04 | 4.79E+04 | 2.14E+03 | 3.91E+04 | 2.65E+04 | 7.17E+04 | 4.58E+04 | 2.33E+04 |
| 981 | 0.6826 | -1.10 | 7.28E+04 | 6.36E+04 | 1.06E+05 | 8.08E+04 | 2.24E+04 | 3.97E+04 | 8.25E+04 | 9.86E+04 | 7.36E+04 | 3.04E+04 |
| 1206 | 0.6835 | 1.17 | 1.13E+05 | 1.15E+05 | 7.18E+04 | 9.99E+04 | 2.44E+04 | 1.20E+05 | 1.60E+05 | 7.07E+04 | 1.17E+05 | 4.47E+04 |
| 2737 | 0.6836 | 1.05 | 9.12E+04 | 2.21E+05 | 8.72E+04 | 1.33E+05 | 7.61E+04 | 1.64E+05 | 1.24E+05 | 1.31E+05 | 1.40E+05 | 2.14E+04 |
| 927 | 0.6861 | -1.07 | 2.31E+05 | 2.49E+05 | 1.69E+05 | 2.16E+05 | 4.21E+04 | 2.34E+05 | 1.92E+05 | 1.79E+05 | 2.02E+05 | 2.88E+04 |
| 2773 | 0.6866 | 1.03 | 1.41E+06 | 1.45E+06 | 1.38E+06 | 1.41E+06 | 3.14E+04 | 1.64E+06 | 1.33E+06 | 1.41E+06 | 1.46E+06 | 1.63E+05 |
| 626 | 0.6884 | 1.04 | 3.70E+04 | 4.00E+04 | 4.82E+04 | 4.17E+04 | 5.81E+03 | 3.90E+04 | 4.45E+04 | 4.64E+04 | 4.33E+04 | 3.82E+03 |
| 1875 | 0.6890 | -1.08 | 7.49E+04 | 5.35E+04 | 7.92E+04 | 6.92E+04 | 1.38E+04 | 5.60E+04 | 8.72E+04 | 4.94E+04 | 6.42E+04 | 2.02E+04 |
| 1332 | 0.6896 | 1.03 | 6.50E+05 | 6.80E+05 | 5.93E+05 | 6.41E+05 | 4.43E+04 | 7.32E+05 | 6.31E+05 | 6.19E+05 | 6.61E+05 | 6.17E+04 |
| 2558 | 0.6938 | -1.14 | 1.87E+06 | 1.64E+06 | 9.37E+05 | 1.48E+06 | 4.86E+05 | 1.18E+06 | 1.47E+06 | 1.26E+06 | 1.30E+06 | 1.48E+05 |
| 3738 | 0.6953 | -1.17 | 2.07E+05 | 2.57E+05 | 1.07E+05 | 1.91E+05 | 7.63E+04 | 2.20E+05 | 9.29E+04 | 1.76E+05 | 1.63E+05 | 6.44E+04 |
| 2826 | 0.6961 | -1.27 | 7.26E+05 | 9.24E+05 | 2.72E+05 | 6.41E+05 | 3.34E+05 | 6.91E+05 | 5.78E+05 | 2.46E+05 | 5.05E+05 | 2.31E+05 |
| 1234 | 0.6985 | -1.07 | 1.75E+05 | 1.84E+05 | 2.49E+05 | 2.03E+05 | 4.04E+04 | 1.39E+05 | 1.65E+05 | 2.65E+05 | 1.90E+05 | 6.67E+04 |
| 1189 | 0.6997 | -1.04 | 1.03E+06 | 1.07E+06 | 8.46E+05 | 9.81E+05 | 1.20E+05 | 1.00E+06 | 9.46E+05 | 8.87E+05 | 9.45E+05 | 5.75E+04 |
| 3719 | 0.7011 | 1.06 | 6.79E+05 | 7.92E+05 | 5.50E+05 | 6.74E+05 | 1.21E+05 | 7.92E+05 | 5.76E+05 | 7.78E+05 | 7.15E+05 | 1.21E+05 |
| 487 | 0.7019 | -1.09 | 1.07E+05 | 1.19E+05 | 1.77E+05 | 1.34E+05 | 3.79E+04 | 9.01E+04 | 1.04E+05 | 1.75E+05 | 1.23E+05 | 4.53E+04 |
| 5131 | 0.7075 | 1.11 | 5.04E+04 | 8.53E+04 | 6.78E+04 | 6.78E+04 | 1.75E+04 | 8.00E+04 | 5.16E+04 | 9.37E+04 | 7.51E+04 | 2.15E+04 |
| 2717 | 0.7102 | 1.16 | 7.66E+04 | 1.00E+05 | 1.03E+05 | 9.31E+04 | 1.44E+04 | 1.12E+05 | 6.54E+04 | 1.46E+05 | 1.08E+05 | 4.04E+04 |
| 405 | 0.7143 | 1.54 | 4.52E+04 | 5.91E+04 | 1.03E+05 | 6.90E+04 | 3.00E+04 | 5.91E+04 | 4.12E+04 | 2.19E+05 | 1.06E+05 | 9.79E+04 |
| 5134 | 0.7243 | -1.04 | 5.73E+05 | 5.66E+05 | 7.01E+05 | 6.13E+05 | 7.58E+04 | 4.82E+05 | 7.19E+05 | 5.63E+05 | 5.88E+05 | 1.21E+05 |
| 383 | 0.7257 | 1.04 | 3.09E+04 | 4.00E+04 | 5.65E+04 | 4.25E+04 | 1.30E+04 | 4.37E+04 | 4.26E+04 | 4.58E+04 | 4.40E+04 | 1.59E+03 |
| 2775 | 0.7260 | -1.13 | 2.71E+05 | 3.41E+05 | 1.66E+05 | 2.60E+05 | 8.83E+04 | 1.87E+05 | 2.59E+05 | 2.44E+05 | 2.30E+05 | 3.80E+04 |
| 2822 | 0.7273 | 1.04 | 6.60E+05 | 8.15E+05 | 6.16E+05 | 6.97E+05 | 1.05E+05 | 7.12E+05 | 8.18E+05 | 6.43E+05 | 7.24E+05 | 8.84E+04 |
| 1215 | 0.7276 | -1.07 | 3.41E+05 | 3.79E+05 | 2.45E+05 | 3.22E+05 | 6.88E+04 | 3.60E+05 | 3.41E+05 | 2.00E+05 | 3.00E+05 | 8.77E+04 |
| 5157 | 0.7341 | -1.00 | 7.08E+05 | 6.72E+05 | 1.81E+05 | 5.20E+05 | 2.95E+05 | 5.18E+05 | 4.97E+05 | 5.45E+05 | 5.20E+05 | 2.40E+04 |
| 2674 | 0.7353 | 1.04 | 5.14E+04 | 6.28E+04 | 3.51E+04 | 4.98E+04 | 1.39E+04 | 5.52E+04 | 5.36E+04 | 4.65E+04 | 5.18E+04 | 4.62E+03 |
| 1059 | 0.7381 | 1.04 | 2.68E+05 | 2.62E+05 | 1.52E+05 | 2.28E+05 | 6.52E+04 | 2.48E+05 | 2.12E+05 | 2.51E+05 | 2.37E+05 | 2.19E+04 |
| 1817 | 0.7395 | 1.02 | 1.45E+06 | 1.69E+06 | 1.66E+06 | 1.60E+06 | 1.29E+05 | 1.70E+06 | 1.52E+06 | 1.68E+06 | 1.63E+06 | 1.02E+05 |
| 2535 | 0.7402 | -1.08 | 4.51E+05 | 4.90E+05 | 2.97E+05 | 4.13E+05 | 1.03E+05 | 4.33E+05 | 3.55E+05 | 3.56E+05 | 3.82E+05 | 4.49E+04 |
| 468 | 0.7406 | -1.12 | 4.75E+04 | 3.40E+04 | 7.63E+04 | 5.26E+04 | 2.16E+04 | 5.62E+04 | 6.04E+04 | 2.44E+04 | 4.70E+04 | 1.97E+04 |
| 2101 | 0.7458 | -1.06 | 8.26E+04 | 9.82E+04 | 6.48E+04 | 8.19E+04 | 1.67E+04 | 1.09E+05 | 5.55E+04 | 6.82E+04 | 7.76E+04 | 2.80E+04 |
| 663 | 0.7519 | -1.02 | 1.99E+05 | 1.80E+05 | 2.19E+05 | 1.99E+05 | 1.92E+04 | 1.63E+05 | 2.96E+05 | 1.24E+05 | 1.95E+05 | 9.02E+04 |
| 5129 | 0.7544 | -1.04 | 1.57E+05 | 1.89E+05 | 2.07E+05 | 1.84E+05 | 2.53E+04 | 1.81E+05 | 1.11E+05 | 2.41E+05 | 1.78E+05 | 6.50E+04 |
| 1423 | 0.7568 | -1.10 | 3.29E+04 | 2.39E+04 | 4.89E+04 | 3.53E+04 | 1.26E+04 | 3.14E+04 | 4.32E+04 | 2.13E+04 | 3.20E+04 | 1.09E+04 |
| 2199 | 0.7584 | 1.05 | 5.43E+04 | 5.49E+04 | 4.67E+04 | 5.20E+04 | 4.57E+03 | 4.53E+04 | 5.24E+04 | 6.61E+04 | 5.46E+04 | 1.06E+04 |
| 3523 | 0.7584 | 1.07 | 2.86E+05 | 1.80E+05 | 1.21E+05 | 1.95E+05 | 8.35E+04 | 1.80E+05 | 2.86E+05 | 1.62E+05 | 2.10E+05 | 6.69E+04 |
| 1741 | 0.7589 | 1.02 | 2.01E+05 | 3.91E+05 | 1.85E+05 | 2.59E+05 | 1.14E+05 | 2.90E+05 | 2.58E+05 | 2.46E+05 | 2.65E+05 | 2.29E+04 |
| 2272 | 0.7621 | -1.03 | 2.38E+05 | 2.44E+05 | 1.98E+05 | 2.27E+05 | 2.48E+04 | 2.48E+05 | 1.83E+05 | 2.27E+05 | 2.19E+05 | 3.28E+04 |
| 424 | 0.7634 | -1.09 | 5.58E+04 | 1.15E+05 | 1.05E+05 | 9.18E+04 | 3.16E+04 | 5.83E+04 | 6.35E+04 | 1.30E+05 | 8.40E+04 | 4.02E+04 |
| 1744 | 0.7639 | 1.04 | 1.51E+05 | 2.08E+05 | 1.05E+05 | 1.55E+05 | 5.18E+04 | 1.84E+05 | 1.51E+05 | 1.46E+05 | 1.60E+05 | 2.07E+04 |
| 1764 | 0.7655 | -1.05 | 6.94E+05 | 7.31E+05 | 5.26E+05 | 6.50E+05 | 1.09E+05 | 7.01E+05 | 6.30E+05 | 5.34E+05 | 6.22E+05 | 8.37E+04 |
| 738 | 0.7700 | 1.03 | 1.74E+05 | 1.80E+05 | 9.43E+04 | 1.49E+05 | 4.76E+04 | 1.45E+05 | 1.58E+05 | 1.56E+05 | 1.53E+05 | 7.17E+03 |
| 4070 | 0.7750 | -1.04 | 3.99E+05 | 3.71E+05 | 2.91E+05 | 3.54E+05 | 5.62E+04 | 4.30E+05 | 2.46E+05 | 3.43E+05 | 3.40E+05 | 9.18E+04 |
| 1236 | 0.7769 | -1.02 | 9.41E+04 | 9.83E+04 | 1.10E+05 | 1.01E+05 | 8.30E+03 | 8.38E+04 | 6.62E+04 | 1.47E+05 | 9.91E+04 | 4.27E+04 |
| 1404 | 0.7797 | -1.04 | 4.58E+04 | 4.88E+04 | 6.40E+04 | 5.28E+04 | 9.79E+03 | 5.17E+04 | 6.44E+04 | 3.58E+04 | 5.07E+04 | 1.43E+04 |
| 1086 | 0.7799 | 1.20 | 4.24E+04 | 4.33E+04 | 3.10E+04 | 3.89E+04 | 6.83E+03 | 3.55E+04 | 2.79E+04 | 7.67E+04 | 4.67E+04 | 2.62E+04 |
| 2943 | 0.7801 | -1.05 | 6.83E+04 | 9.57E+04 | 6.88E+04 | 7.76E+04 | 1.57E+04 | 7.32E+04 | 8.04E+04 | 6.85E+04 | 7.40E+04 | 5.96E+03 |
| 3950 | 0.7805 | -1.03 | 2.79E+05 | 3.00E+05 | 2.26E+05 | 2.68E+05 | 3.82E+04 | 2.63E+05 | 2.44E+05 | 2.72E+05 | 2.60E+05 | 1.44E+04 |
| 5126 | 0.7821 | 1.17 | 3.05E+05 | 2.37E+05 | 2.23E+05 | 2.55E+05 | 4.39E+04 | 3.20E+05 | 4.19E+05 | 1.58E+05 | 2.99E+05 | 1.32E+05 |
| 4182 | 0.7823 | 1.06 | 7.70E+05 | 7.53E+05 | 4.76E+05 | 6.66E+05 | 1.65E+05 | 8.93E+05 | 5.52E+05 | 6.74E+05 | 7.06E+05 | 1.72E+05 |
| 5099 | 0.7824 | -1.04 | 3.93E+04 | 4.18E+04 | 2.99E+04 | 3.70E+04 | 6.27E+03 | 3.58E+04 | 3.74E+04 | 3.34E+04 | 3.55E+04 | 2.03E+03 |
| 1635 | 0.7862 | 1.09 | 5.12E+04 | 7.65E+04 | 4.88E+04 | 5.88E+04 | 1.54E+04 | 7.47E+04 | 4.14E+04 | 7.65E+04 | 6.42E+04 | 1.98E+04 |
| 2785 | 0.7888 | -1.14 | 1.03E+05 | 2.55E+05 | 1.68E+05 | 1.75E+05 | 7.60E+04 | 1.91E+05 | 1.07E+05 | 1.66E+05 | 1.54E+05 | 4.32E+04 |
| 636 | 0.7900 | 1.03 | 1.27E+05 | 1.31E+05 | 1.12E+05 | 1.23E+05 | 1.01E+04 | 1.46E+05 | 1.26E+05 | 1.09E+05 | 1.27E+05 | 1.85E+04 |
| 245 | 0.7958 | 1.05 | 7.02E+04 | 8.70E+04 | 1.08E+05 | 8.85E+04 | 1.90E+04 | 9.28E+04 | 1.11E+05 | 7.38E+04 | 9.26E+04 | 1.87E+04 |
| 1275 | 0.7985 | 1.06 | 4.48E+05 | 6.54E+05 | 5.00E+05 | 5.34E+05 | 1.07E+05 | 5.96E+05 | 4.12E+05 | 6.97E+05 | 5.68E+05 | 1.44E+05 |
| 635 | 0.8002 | -1.03 | 1.17E+05 | 1.30E+05 | 9.61E+04 | 1.14E+05 | 1.69E+04 | 1.34E+05 | 1.09E+05 | 8.88E+04 | 1.11E+05 | 2.26E+04 |
| 1736 | 0.8034 | 1.02 | 2.06E+05 | 2.17E+05 | 1.58E+05 | 1.94E+05 | 3.13E+04 | 2.18E+05 | 1.79E+05 | 1.99E+05 | 1.99E+05 | 1.94E+04 |
| 5101 | 0.8050 | 1.02 | 2.31E+04 | 1.89E+04 | 3.41E+04 | 2.54E+04 | 7.84E+03 | 2.92E+04 | 2.36E+04 | 2.50E+04 | 2.59E+04 | 2.94E+03 |
| 2229 | 0.8064 | -1.05 | 2.55E+05 | 2.47E+05 | 1.47E+05 | 2.17E+05 | 6.04E+04 | 1.18E+05 | 2.13E+05 | 2.88E+05 | 2.06E+05 | 8.51E+04 |
| 2183 | 0.8092 | -1.02 | 6.22E+05 | 5.55E+05 | 3.49E+05 | 5.09E+05 | 1.42E+05 | 2.24E+05 | 5.49E+05 | 7.21E+05 | 4.98E+05 | 2.52E+05 |
| 4243 | 0.8095 | -1.13 | 3.98E+05 | 4.22E+05 | 1.37E+05 | 3.19E+05 | 1.58E+05 | 1.81E+05 | 1.80E+05 | 4.82E+05 | 2.81E+05 | 1.74E+05 |
| 429 | 0.8101 | -1.17 | 2.66E+04 | 6.31E+04 | 9.74E+04 | 6.24E+04 | 3.54E+04 | 3.71E+04 | 3.42E+04 | 8.85E+04 | 5.33E+04 | 3.05E+04 |
| 430 | 0.8164 | 1.31 | 7.20E+04 | 1.00E+05 | 1.14E+05 | 9.55E+04 | 2.15E+04 | 5.84E+04 | 8.35E+04 | 2.34E+05 | 1.25E+05 | 9.49E+04 |
| 379 | 0.8205 | -1.04 | 6.12E+04 | 6.78E+04 | 4.57E+04 | 5.82E+04 | 1.14E+04 | 6.14E+04 | 5.98E+04 | 4.63E+04 | 5.58E+04 | 8.33E+03 |
| 734 | 0.8303 | 1.03 | 1.45E+05 | 1.32E+05 | 9.29E+04 | 1.23E+05 | 2.70E+04 | 1.14E+05 | 1.48E+05 | 1.17E+05 | 1.27E+05 | 1.89E+04 |
| 3036 | 0.8319 | 1.13 | 4.63E+04 | 6.36E+04 | 7.49E+04 | 6.16E+04 | 1.44E+04 | 8.01E+04 | 3.75E+04 | 9.08E+04 | 6.95E+04 | 2.82E+04 |
| 1264 | 0.8329 | 1.01 | 4.03E+05 | 3.46E+05 | 3.35E+05 | 3.61E+05 | 3.64E+04 | 3.54E+05 | 3.54E+05 | 3.88E+05 | 3.65E+05 | 1.97E+04 |
| 940 | 0.8358 | 1.02 | 4.64E+05 | 4.18E+05 | 2.79E+05 | 3.87E+05 | 9.64E+04 | 4.62E+05 | 3.78E+05 | 3.48E+05 | 3.96E+05 | 5.94E+04 |
| 1751 | 0.8468 | 1.09 | 2.12E+05 | 2.08E+05 | 1.71E+05 | 1.97E+05 | 2.27E+04 | 2.11E+05 | 2.98E+05 | 1.39E+05 | 2.16E+05 | 7.94E+04 |
| 797 | 0.8546 | -1.05 | 2.43E+05 | 1.51E+05 | 1.35E+05 | 1.76E+05 | 5.79E+04 | 2.04E+05 | 1.90E+05 | 1.10E+05 | 1.68E+05 | 5.04E+04 |
| 456 | 0.8586 | 1.06 | 4.92E+04 | 6.72E+04 | 8.05E+04 | 6.56E+04 | 1.57E+04 | 6.98E+04 | 4.75E+04 | 9.18E+04 | 6.97E+04 | 2.22E+04 |
| 1464 | 0.8612 | -1.03 | 2.33E+05 | 2.55E+05 | 1.87E+05 | 2.25E+05 | 3.47E+04 | 2.48E+05 | 1.95E+05 | 2.16E+05 | 2.19E+05 | 2.68E+04 |
| 455 | 0.8617 | -1.11 | 2.25E+04 | 3.76E+04 | 5.62E+04 | 3.87E+04 | 1.69E+04 | 3.33E+04 | 2.72E+04 | 4.44E+04 | 3.50E+04 | 8.74E+03 |
| 3017 | 0.8624 | -1.08 | 1.45E+05 | 2.68E+05 | 1.47E+05 | 1.87E+05 | 7.05E+04 | 1.61E+05 | 1.45E+05 | 2.14E+05 | 1.74E+05 | 3.64E+04 |
| 5093 | 0.8652 | 1.02 | 1.72E+05 | 1.67E+05 | 1.13E+05 | 1.50E+05 | 3.28E+04 | 1.76E+05 | 1.60E+05 | 1.25E+05 | 1.54E+05 | 2.60E+04 |
| 2864 | 0.8667 | 1.09 | 4.48E+05 | 5.24E+05 | 2.42E+05 | 4.05E+05 | 1.46E+05 | 8.17E+05 | 3.60E+05 | 1.43E+05 | 4.40E+05 | 3.44E+05 |
| 3208 | 0.8707 | 1.01 | 1.75E+05 | 2.12E+05 | 1.58E+05 | 1.82E+05 | 2.75E+04 | 1.85E+05 | 1.97E+05 | 1.70E+05 | 1.84E+05 | 1.33E+04 |
| 5112 | 0.8713 | -1.01 | 1.43E+06 | 1.65E+06 | 1.40E+06 | 1.49E+06 | 1.36E+05 | 1.54E+06 | 1.63E+06 | 1.25E+06 | 1.47E+06 | 1.95E+05 |
| 2046 | 0.8747 | 1.02 | 3.69E+05 | 3.52E+05 | 2.58E+05 | 3.26E+05 | 5.97E+04 | 2.87E+05 | 3.95E+05 | 3.19E+05 | 3.34E+05 | 5.55E+04 |
| 2940 | 0.8793 | -1.01 | 1.78E+06 | 2.03E+06 | 1.01E+06 | 1.61E+06 | 5.30E+05 | 1.50E+06 | 1.69E+06 | 1.60E+06 | 1.60E+06 | 9.47E+04 |
| 905 | 0.8831 | -1.01 | 4.53E+04 | 4.79E+04 | 4.75E+04 | 4.69E+04 | 1.38E+03 | 4.20E+04 | 5.06E+04 | 4.73E+04 | 4.66E+04 | 4.31E+03 |
| 2168 | 0.8907 | -1.08 | 1.14E+05 | 1.09E+05 | 5.62E+04 | 9.32E+04 | 3.22E+04 | 8.21E+04 | 1.02E+05 | 7.50E+04 | 8.64E+04 | 1.41E+04 |
| 1644 | 0.8908 | 1.00 | 1.88E+05 | 1.82E+05 | 2.06E+05 | 1.92E+05 | 1.25E+04 | 1.65E+05 | 1.50E+05 | 2.63E+05 | 1.93E+05 | 6.17E+04 |
| 630 | 0.8947 | -1.03 | 9.44E+04 | 8.32E+04 | 6.17E+04 | 7.98E+04 | 1.66E+04 | 8.63E+04 | 8.23E+04 | 6.39E+04 | 7.75E+04 | 1.19E+04 |
| 1258 | 0.8954 | -1.06 | 9.73E+05 | 1.57E+06 | 5.98E+05 | 1.05E+06 | 4.91E+05 | 1.51E+06 | 8.89E+05 | 5.79E+05 | 9.91E+05 | 4.72E+05 |
| 431 | 0.8961 | 1.22 | 4.66E+04 | 7.50E+04 | 7.57E+04 | 6.58E+04 | 1.66E+04 | 4.47E+04 | 4.73E+04 | 1.50E+05 | 8.05E+04 | 5.98E+04 |
| 3169 | 0.8967 | 1.01 | 3.17E+05 | 6.62E+05 | 4.09E+05 | 4.63E+05 | 1.79E+05 | 5.68E+05 | 3.52E+05 | 4.76E+05 | 4.66E+05 | 1.08E+05 |
| 1318 | 0.8989 | -1.01 | 3.96E+05 | 4.13E+05 | 3.50E+05 | 3.86E+05 | 3.22E+04 | 3.80E+05 | 3.17E+05 | 4.54E+05 | 3.84E+05 | 6.86E+04 |
| 647 | 0.9018 | 1.01 | 3.60E+05 | 2.55E+05 | 3.46E+05 | 3.20E+05 | 5.71E+04 | 3.43E+05 | 3.47E+05 | 2.81E+05 | 3.23E+05 | 3.73E+04 |
| 2769 | 0.9036 | 1.04 | 1.83E+05 | 1.47E+05 | 1.49E+05 | 1.59E+05 | 2.05E+04 | 2.17E+05 | 1.40E+05 | 1.40E+05 | 1.66E+05 | 4.43E+04 |
| 755 | 0.9050 | 1.02 | 6.92E+05 | 6.90E+05 | 5.89E+05 | 6.57E+05 | 5.90E+04 | 7.68E+05 | 5.89E+05 | 6.44E+05 | 6.67E+05 | 9.18E+04 |
| 2361 | 0.9064 | -1.03 | 4.63E+04 | 4.58E+04 | 3.02E+04 | 4.08E+04 | 9.15E+03 | 3.52E+04 | 3.95E+04 | 4.35E+04 | 3.94E+04 | 4.14E+03 |
| 1645 | 0.9080 | -1.01 | 1.97E+05 | 2.18E+05 | 2.71E+05 | 2.29E+05 | 3.80E+04 | 1.90E+05 | 2.10E+05 | 2.77E+05 | 2.26E+05 | 4.54E+04 |
| 762 | 0.9140 | 1.02 | 7.40E+05 | 7.43E+05 | 5.63E+05 | 6.82E+05 | 1.03E+05 | 8.26E+05 | 6.21E+05 | 6.31E+05 | 6.93E+05 | 1.16E+05 |
| 741 | 0.9162 | 1.02 | 4.31E+05 | 4.31E+05 | 3.71E+05 | 4.11E+05 | 3.45E+04 | 4.82E+05 | 4.12E+05 | 3.59E+05 | 4.18E+05 | 6.14E+04 |
| 1050 | 0.9198 | -1.06 | 1.37E+05 | 9.17E+04 | 6.64E+04 | 9.84E+04 | 3.59E+04 | 7.70E+04 | 1.09E+05 | 9.23E+04 | 9.28E+04 | 1.61E+04 |
| 3002 | 0.9202 | 1.06 | 1.23E+05 | 1.21E+05 | 9.32E+04 | 1.12E+05 | 1.67E+04 | 1.67E+05 | 5.46E+04 | 1.36E+05 | 1.19E+05 | 5.80E+04 |
| 5140 | 0.9213 | 1.05 | 2.41E+05 | 2.32E+05 | 2.80E+05 | 2.51E+05 | 2.55E+04 | 3.40E+05 | 3.27E+05 | 1.27E+05 | 2.64E+05 | 1.19E+05 |
| 1774 | 0.9224 | 1.06 | 3.68E+05 | 3.11E+05 | 3.95E+05 | 3.58E+05 | 4.28E+04 | 4.49E+05 | 5.19E+05 | 1.74E+05 | 3.81E+05 | 1.82E+05 |
| 2816 | 0.9251 | 1.07 | 5.21E+05 | 4.94E+05 | 4.39E+05 | 4.84E+05 | 4.20E+04 | 3.91E+05 | 4.03E+05 | 7.68E+05 | 5.20E+05 | 2.14E+05 |
| 643 | 0.9291 | 1.07 | 6.41E+04 | 5.11E+04 | 6.43E+04 | 5.99E+04 | 7.57E+03 | 7.37E+04 | 3.85E+04 | 7.96E+04 | 6.40E+04 | 2.23E+04 |
| 2189 | 0.9467 | 1.02 | 2.49E+05 | 2.14E+05 | 9.72E+04 | 1.87E+05 | 7.95E+04 | 9.71E+04 | 1.50E+05 | 3.23E+05 | 1.90E+05 | 1.18E+05 |
| 1445 | 0.9494 | 1.02 | 7.76E+05 | 5.89E+05 | 3.80E+05 | 5.82E+05 | 1.98E+05 | 5.00E+05 | 8.35E+05 | 4.41E+05 | 5.92E+05 | 2.12E+05 |
| 2610 | 0.9502 | 1.05 | 2.21E+06 | 2.58E+06 | 1.76E+06 | 2.18E+06 | 4.12E+05 | 3.09E+06 | 2.40E+06 | 1.43E+06 | 2.30E+06 | 8.34E+05 |
| 2564 | 0.9521 | 1.02 | 5.71E+05 | 3.52E+05 | 3.36E+05 | 4.20E+05 | 1.31E+05 | 6.02E+05 | 3.47E+05 | 3.40E+05 | 4.30E+05 | 1.49E+05 |
| 1437 | 0.9552 | -1.03 | 1.98E+05 | 1.90E+05 | 1.14E+05 | 1.67E+05 | 4.63E+04 | 1.77E+05 | 1.79E+05 | 1.30E+05 | 1.62E+05 | 2.74E+04 |
| 1455 | 0.9661 | -1.01 | 7.35E+04 | 6.32E+04 | 1.20E+05 | 8.56E+04 | 3.04E+04 | 6.97E+04 | 7.60E+04 | 1.09E+05 | 8.48E+04 | 2.10E+04 |
| 1185 | 0.9689 | -1.02 | 4.52E+05 | 5.11E+05 | 3.24E+05 | 4.29E+05 | 9.54E+04 | 4.54E+05 | 4.08E+05 | 3.97E+05 | 4.20E+05 | 3.04E+04 |
| 1312 | 0.9715 | 1.02 | 3.18E+05 | 3.15E+05 | 2.62E+05 | 2.98E+05 | 3.15E+04 | 3.75E+05 | 2.79E+05 | 2.54E+05 | 3.03E+05 | 6.38E+04 |
| 2554 | 0.9799 | -1.03 | 4.83E+05 | 3.05E+05 | 2.53E+05 | 3.47E+05 | 1.21E+05 | 3.24E+05 | 3.46E+05 | 3.38E+05 | 3.36E+05 | 1.09E+04 |
| 1569 | 0.9857 | -1.01 | 1.95E+06 | 1.98E+06 | 1.49E+06 | 1.81E+06 | 2.76E+05 | 1.97E+06 | 1.59E+06 | 1.82E+06 | 1.79E+06 | 1.87E+05 |
| 2755 | 0.9860 | -1.02 | 1.28E+05 | 1.72E+05 | 1.04E+05 | 1.35E+05 | 3.45E+04 | 1.38E+05 | 1.35E+05 | 1.23E+05 | 1.32E+05 | 7.53E+03 |
| 1281 | 0.9881 | -1.01 | 3.51E+05 | 2.67E+05 | 2.12E+05 | 2.77E+05 | 7.01E+04 | 2.10E+05 | 2.76E+05 | 3.40E+05 | 2.75E+05 | 6.47E+04 |
| 432 | 0.9938 | 1.02 | 6.68E+04 | 9.43E+04 | 1.65E+05 | 1.09E+05 | 5.07E+04 | 7.11E+04 | 7.97E+04 | 1.81E+05 | 1.11E+05 | 6.14E+04 |
| 3410 | 0.9971 | -1.02 | 6.88E+04 | 5.91E+04 | 1.01E+05 | 7.62E+04 | 2.18E+04 | 8.72E+04 | 7.63E+04 | 6.16E+04 | 7.51E+04 | 1.28E+04 |
| 3740 | 0.9998 | -1.01 | 3.46E+05 | 3.81E+05 | 2.00E+05 | 3.09E+05 | 9.63E+04 | 3.64E+05 | 2.12E+05 | 3.41E+05 | 3.06E+05 | 8.19E+04 |
